# Triplet Sensitization Photon Upconversion Using Near-Infrared Indirect-Bandgap AgBiS_2_ Nanocrystals

**DOI:** 10.1021/jacs.5c04015

**Published:** 2025-04-09

**Authors:** Kin Ting Chang, Wenfei Liang, Shaokuan Gong, Pang Ho Yeung, Jianning Feng, Xihan Chen, Haipeng Lu

**Affiliations:** † Department of Chemistry, The Hong Kong University of Science and Technology, Clear Water Bay, Kowloon, Hong Kong 999077, China (SAR); ‡ Department of Mechanical and Energy Engineering, Southern University of Science and Technology, Shenzhen, Guangdong 518055, China

## Abstract

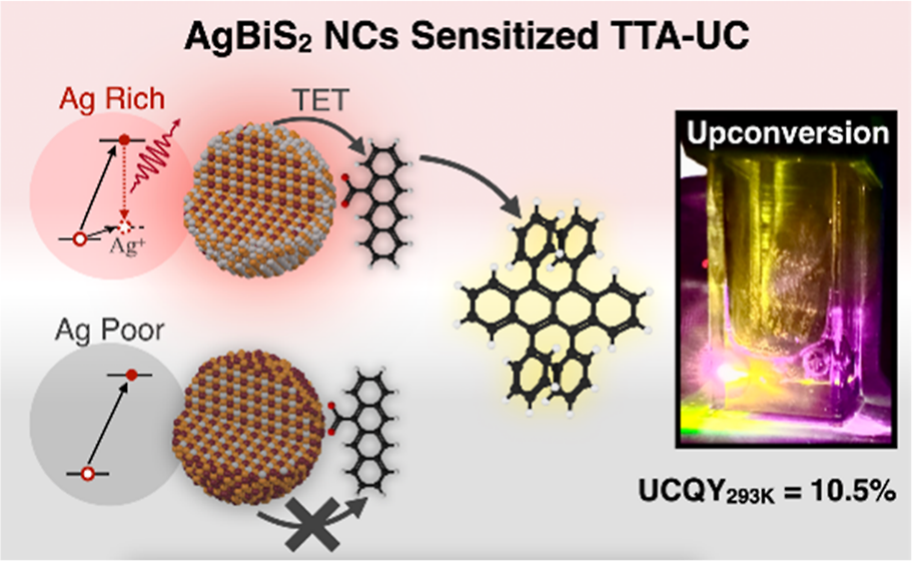

Colloidal semiconductor nanocrystals (NCs) have recently emerged as ideal triplet sensitizers owing to their diverse material composition and spectral tunability. However, the NCs that can efficiently sensitize near-infrared-to-visible photon upconversion remain largely limited to toxic lead-based NCs. Here, we present a new lead-free, near-infrared, indirect-bandgap sensitizer based on AgBiS_2_ NCs, enabling near-infrared-to-yellow upconversion with a quantum yield reaching 10.5% (normalized to 100%). The key to success is the precise stoichiometry control of AgBiS_2_ NCs, which provides the essential surface states for both radiative recombination and triplet energy transfer. Ultrafast transient absorption spectroscopy verifies the efficient triplet energy transfer mechanism mediated by surface states. Our work presents a new eco-friendly material system for efficient triplet fusion near-infrared photon upconversion.

## Introduction

Infrared colloidal semiconductor nanocrystals (NCs) are promising candidates for infrared-active optoelectronic devices, including photovoltaics, infrared light-emitting diodes, telecommunications, and in vivo imaging.^1^ More recently, near-infrared (NIR) semiconductor NCs have emerged as an efficient triplet sensitizer to enable NIR-to-visible photon upconversion (UC) via triplet–triplet annihilation photon UC (TTA-UC). Since the first report by Tang and Bardeen^2^ on the NIR-to-visible photon UC based on PbSe NCs, the field has witnessed tremendous progress.^2−5^ However, the NCs that can efficiently sensitize NIR-to-visible UC remain limited, partly due to the underdevelopment of NIR NCs. For a long time, the only family of NCs capable of sensitizing NIR-to-visible TTA-UC was lead chalcogenide NCs. The use of nontoxic, lead-free, NIR NCs for TTA-UC has been notably lacking until two recent studies emerged. In 2023, Wu’s group reported an efficient NIR-to-visible TTA-UC system using CuInSe_2_ NCs, achieving an UC quantum yield (UCQY) of 16.7% (normalized to 100%).^6^ However, CuInSe_2_ NCs require a full optimization of stoichiometry, doping, and core/shell structure to obtain efficient TTA-UC. The use of Se also raises significant environmental and health concerns as Se is generally regarded a toxic element. More recently, Ji’s group achieved a record NIR-to-visible TTA-UC UCQY of 21% (normalized to 100%) with InAs NCs as the triplet sensitizer,^7^ surpassing previous demonstrations based on molecular sensitizers containing precious metals. Nevertheless, the synthesis of high-quality colloidal InAs NCs requires high temperature (>350 °C) and the use of extremely toxic, highly reactive precursor tris(trimethylsilyl)arsine. These works highlight an exciting and urgent direction for exploring nontoxic NIR NCs to convert photons beyond the Si bandgap.

Recently, colloidal AgBiS_2_ NCs have emerged as a promising nontoxic NIR material for optoelectronic devices (Note S1). The synthesis of AgBiS_2_ NCs appears to be notably straightforward and only requires low temperatures (around 100 °C). In 2016, Konstantatos reported the first solution-processed AgBiS_2_ NCs solar cell, achieving a notable power conversion efficiency (PCE) of 6.17%.^8^ Johansson showed that the stoichiometry of AgBiS_2_ NCs plays a crucial role in the solar cell performance, and silver-deficient composition yielded an optimal PCE.^9^ More recently, Konstantatos optimized the cation disorder homogeneity in AgBiS_2_ NCs, achieving a certified PCE of 8.85%.^10^ However, the application of AgBiS_2_ NCs has largely been limited to photovoltaic devices,^11^ and NIR luminescence has rarely been reported, likely due to the indirect bandgap nature. Thus far, the use of indirect-bandgap semiconductors for triplet sensitization in photon UC is largely unexplored, with the only exception of silicon NCs^12^ which upconvert green and red photons to violet photons. However, development of indirect-bandgap NIR NCs that can upconvert NIR photons to visible photons remains essentially unknown.

In this work, we systematically varied the synthetic conditions to explore the relationship between stoichiometry and the optical properties of AgBiS_2_ NCs. Interestingly, we found that a silver-excess stoichiometry can induce the NIR emission in AgBiS_2_ NCs. Spectroscopic studies showed that the NIR emission arises from the recombination of a photogenerated electron and a hole trapped by the excess silver. These NIR-emissive AgBiS_2_ NCs can then be used to sensitize TTA-UC, converting 808 nm NIR photons to 567 nm visible photons with an UCQY of 10.5% (normalized to 100%) at room temperature. The UCQY can be further improved to 17% (normalized to 100%) at 200 K by reducing the nonradiative pathways. Transient absorption (TA) studies show that the triplet energy transfer (TET) from NCs to triplet transmitter is mediated through the trap states of AgBiS_2_ NCs. Our findings highlight AgBiS_2_ NCs as a viable, nontoxic, and sustainable alternative to existing NIR materials for upconverting photons beyond the Si bandgap.

## Results

### Stoichiometry-Dependent NIR Emission of AgBiS_2_ NCs

**Figure 1:**
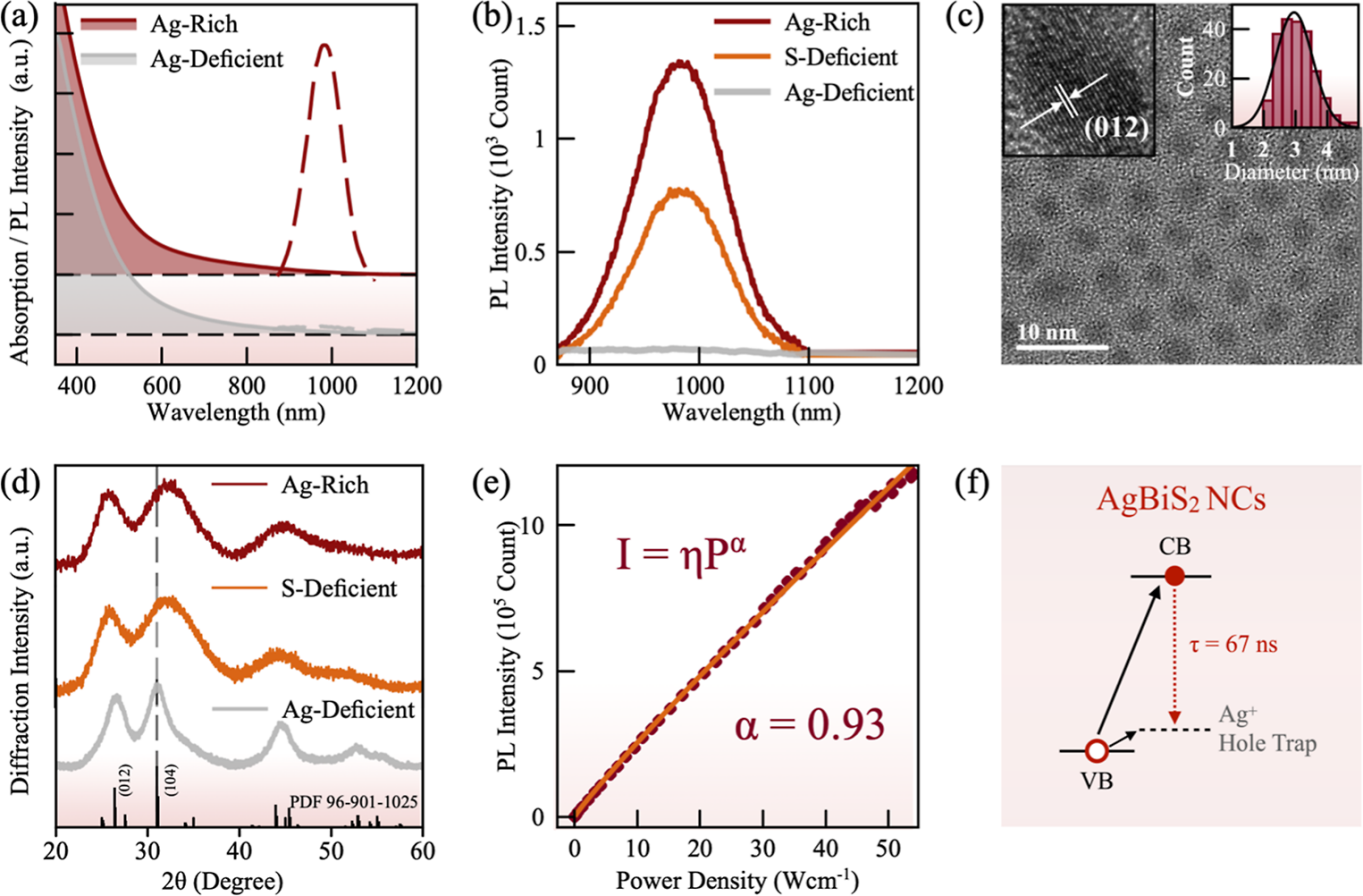
(a) Absorption (solid lines) and PL (dashed lines) spectra of silver-deficient (0.8:1:1, gray) and silver-rich (1.2:1:1, red) AgBiS_2_ NCs. (b) PL spectra of AgBiS_2_ NCs with different Ag/Bi/S feed ratios. (c) TEM image of the optimized silver-rich AgBiS_2_ NCs. The inset shows the size-distribution histogram and HRTEM image. (d) Powder XRD patterns of AgBiS_2_ NCs synthesized with different feed ratios: silver-deficient (0.8:1:1, gray), sulfur-deficient (1.1:1:0.85), and silver-rich (1.2:1:1, red). (e) Integrated PL intensity of AgBiS_2_ NCs colloidal solution at different excitation powers of an 808 nm laser. The solid line shows the fitting curve. (f) Schematic of PL emission mechanism in AgBiS_2_ NCs. CB and VB refer to conduction band and valence band of the AgBiS_2_ NCs.

Adapting from the method reported by Öberg et al.,^9^ we synthesized AgBiS_2_ NCs using silver acetate, bismuth acetate, and hexamethyl-disilathiane (HMS), along with oleic acid (OA) ligands (see details in Methods). The Ag/Bi:S ratio was varied by changing the feed ratio of precursors. Figure 1a shows the UV–vis–NIR absorption and photoluminescence (PL) spectra of the as-synthesized AgBiS_2_ NCs with a silver-deficient ratio (0.8:1:1)^10^ (gray) and silver-rich ratio (1.2:1:1) (red). The absorption spectra of both NCs are similar to previously reported colloidal ternary I–V–VI_2_ NCs,^13−15^ with an absorption tail extending up to 1000 nm without excitonic features, consistent with the indirect-bandgap nature. The PL spectra reveal significant differences between the two samples. As shown in Figure 1a, silver-rich AgBiS_2_ NCs display a strong and sharp PL centered at 980 nm upon excitation with an 808 nm laser, whereas silver-deficient AgBiS_2_ NCs do not exhibit any observable PL. However, the PL intensity is highly sensitive to the feed ratio of both Ag/Bi and Bi/S. A sulfur-deficient composition (with a Ag/Bi/S ratio of 1.1:1:0.85, orange) also leads to a lowered PL intensity (Figure 1b). Here we systematically varied the feed ratio of Ag/Bi and Bi/S and plot the PL intensity as a function of feed ratios in a two-dimensional (2D) pseudocolored map (Figure S1). The optimized AgBiS_2_ NCs give a PLQY of ∼0.8% with a Ag/Bi:S ratio of 1.2:1:1. Figure 1c shows the transmission electron microscopy (TEM) image of the optimized AgBiS_2_ NCs, with the inset showing the size-distribution histogram and high-resolution TEM image. The optimized NCs show an average diameter of 2.96 ± 0.68 nm with high crystallinity.

To confirm the phase purity of AgBiS_2_ NCs, powder X-ray diffraction (PXRD) was performed. Figure 1d shows the PXRD patterns of the three representatives AgBiS_2_ NCs discussed in Figure 1b. The PXRD patterns of all three AgBiS_2_ NCs match the reference pattern for the Matildite phase of AgBiS_2_ (PDF 96-901-1025). For sulfur-deficient (1.1:1:0.85) AgBiS_2_ NCs, the (104) peak shifts to a higher angle, indicating a decrease in lattice parameter. This shift is even more pronounced in the silver-rich (1.2:1:1) AgBiS_2_ NCs, as shown in Figure 1d, suggesting a significant lattice contraction with additional Ag^+^ input. Moreover, all of the diffraction peaks broadened gradually by increasing the Ag^+^ feed ratio, indicating a decreased NC size, in agreement with a previous study.^9^ The composition of the NCs was further analyzed using induction-coupled plasma optical emission spectrometry (ICP-OES). Elemental analysis showed that the incorporated Ag^+^ content in the AgBiS_2_ NCs exhibits a direct correlation with the input Ag^+^ content (Table S1). Our optimized silver-rich AgBiS_2_ NCs do not display any impurity phases such as Ag_2_S or Bi_2_S_3_. However, a large excess of silver would lead to the formation of Ag_2_S NCs, which did not exhibit any PL (Figure S2).

It is worth noting that NIR emission is rarely observed in AgBiS_2_ NCs owing to their indirect bandgap nature. To understand the stoichiometry-dependent NIR emission, excitation-intensity-dependent and time-resolved PL (TRPL) spectroscopies were employed. Figure S3 shows the PL spectra of AgBiS_2_ NCs excited by an 808 nm continuous wave (cw) laser with different excitation intensities (0–55 Wcm^–2^). The power-dependent PL intensity can be used to probe the PL mechanism by fitting the curve with the following equation^16^
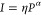
1where *I* is the integrated PL intensity; η is the emission efficiency; *P* is the excitation power density; and α represents the radiative recombination mechanism. Generally, PL is ascribed to free carrier recombination, exciton recombination, and donor–acceptor or free-to-bound exciton recombination for α = 2, 1<α < 2, and α < 1, respectively.^17−21^Figure 1e shows the integrated PL intensity under varying excitation power intensities. Fitting the data with eq 1 yields α to be 0.93 for AgBiS_2_ NCs. The absence of PL peak shift or shape change suggests that the PL originates from a free-to-bound exciton recombination rather than donor–acceptor recombination.^21,22^ Fitting of the TRPL kinetics (Figure S4) gives an average lifetime of 67.1 ns, consistent with PL lifetime of free-to-bound emission in similar systems such as Ag^+^-doped CdSe and CdS NCs.^23−25^ A previous study^26^ has demonstrated that Ag^+^ ions can indeed act as hole traps when doped into CdSe NC host in a similar manner to Cu^+^. Both Ag^+^- and Cu^+^-doped NCs are predicted to exhibit similar localization of photogenerated holes within the [MX_4_] tetrahedron (M = Ag^+^ or Cu^+^, X = chalcogen), highlighting the feasibility of Ag^+^ as the hole trap in our system. Therefore, we conclude that the origin of our PL is similar to what was previously proposed for CuInS_2_/ZnS NCs,^27,28^ that is, a free electron recombines with a hole localized on a deep acceptor level likely associated with the surface Ag^+^ sites (Figure 1f). Furthermore, elemental analysis reveals a strong correlation between Ag^+^ content and PL intensity in AgBiS_2_ NCs (Figure S5). PL intensity initially increases with the Ag^+^ content, but excessive amounts of Ag^+^ lead to a sharp decline in PL intensity. The control of a slight excess of Ag^+^ contents is thus critical to turn on the PL of AgBiS_2_ NCs.

### NIR-to-Visible Photon UC by AgBiS_2_ NC Triplet Sensitization

**Figure 2:**
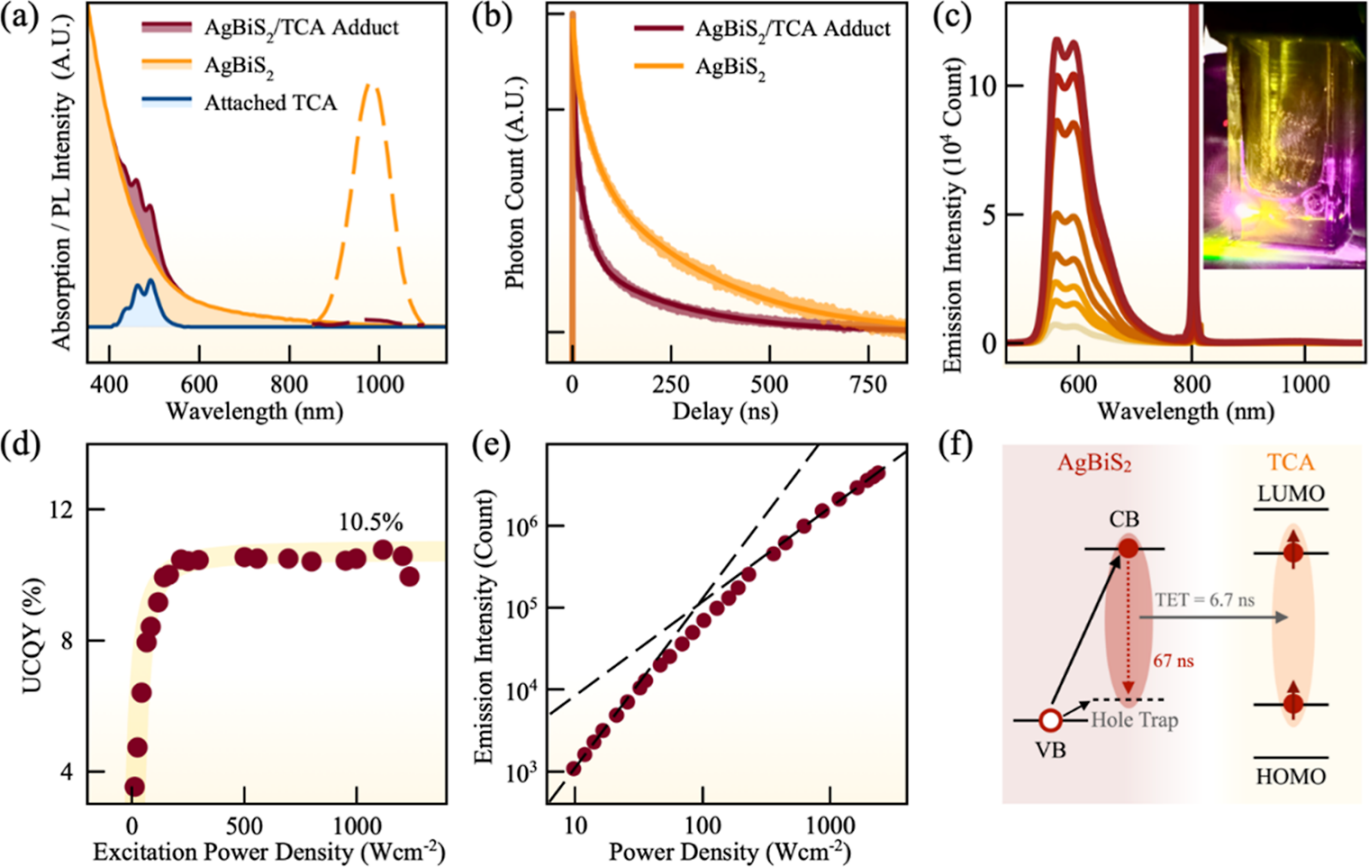
(a) Absorption (solid lines) and PL (dashed lines) spectra of AgBiS_2_ NCs (orange) and AgBiS_2_/TCA(red) dispersed in hexane. The absorption spectra of TCA bound to the surface of AgBiS_2_ NCs (blue) is shown as well. (b) Time-resolved PL decay of AgBiS_2_ NCs (orange) and AgBiS_2_/TCA (red). (c) TTA-UC emission spectra at different excitation powers of an 808 nm laser. Inset: Photograph of NIR-to-visible UC in a nitrogen-filled airtight cuvette, excited by an 808 nm cw-laser. (d) Dependence of normalized UCQY on excitation power. The maximum UCQY achieved is indicated (solid line). (e) TTA-UC intensity as a function of excitation power density of the AgBiS_2_ NC UC system. The crossing point between the low-power quadratic and high-power linear regions is the TTA-UC threshold. (f) Schematic of excited state and energy transfer dynamics of AgBiS_2_ NCs. The triplet state of TCA with energy of ∼1.3 eV is figuratively shown as an intragap level within the energy gap of TCA. CB, VB, LUMO, and HOMO refers to conduction band, valence band, and lowest unoccupied and highest occupied molecular orbitals, respectively.

As the surface Ag^+^ states can serve as the radiative recombination center, we hypothesize that excitons can also transfer their energy through these surface states, similar to what was observed in PbS NCs.^29^ In order to extract triplet energy from AgBiS_2_ NCs, the optimized AgBiS_2_ NC was ligand-exchanged with 5-tetracene carboxylic acid (TCA) with a triplet state of ∼1.3 eV.^30^Figure 2a shows the absorption spectra of AgBiS_2_-TCA and AgBiS_2_ NCs. Ligand-exchanged AgBiS_2_-TCA NCs show three additional absorption peaks in the 400–500 nm region, corresponding to the TCA molecules on the surface of NCs. The surface-attached TCA exhibits a redshift of 46.9 meV when compared to free TCA (Figure S6), an indication of electronic coupling between NCs and TCA. The absorption spectrum of the AgBiS_2_ NCs is not affected by the attachment of TCA molecules. The average number of TCA per NC is estimated to be ∼24 based on their molar extinction coefficient (Figure S7). Upon attaching TCA ligands, the PL intensity of AgBiS_2_ NCs was quenched by ∼98% (Figure 2a), indicating an efficient TET. Furthermore, TRPL spectroscopy (Figure 2b) reveals that the average PL lifetime of AgBiS_2_ NCs was shortened from 67 to 6 ns after TCA ligand exchange. The estimated TET rate was thus determined as 0.15 ns^–1^, with a TET efficiency of 90.8% in the AgBiS_2_-TCA adduct (Note S2).

Encouraged by the exceptionally high TET efficiency at the AgBiS_2_-TCA interface, we then constructed a NIR-to-visible photon UC system, consisting of AgBiS_2_ NCs (sensitizer), surface-attached TCA molecules (transmitter), and rubrene molecules (annihilator) dissolved in toluene. Figure 2c presents a photograph and the emission spectra of the UC system when excited by an 808 nm (cw) laser. The spectra show a clear UC emission in the region of 550–650 nm, consistent with the PL spectra of rubrene molecules.^31^Figure 2d shows the UCQY as a function of the excitation power density, which plateaus at higher excitation powers. The highest UCQY of our system was found to be 10.5% (normalized to 100%). The UC PL intensity shows a quadratic dependence at lower powers and a linear dependence at higher powers, characteristic behaviors of the two-photon TTA-UC process. The crossover point is at 93 W cm^–2^ (Figure 2e), which is defined as the TTA-UC threshold. It is worth noting that AgBiS_2_ NCs is also capable to convert NIR-II photons, that is 980 nm photons, to visible photons with a 0.8% (normalized to 100%) UCQY (Figure S8, Note S3). The PL mechanism of AgBiS_2_ NCs and energy transfer process between the NC and TCA are summarized in Figure 2f.

Interestingly, AgBiS_2_ NCs with no NIR emission also results in no observable TTA-UC in the NC-TCA adduct UC system, indicating the essential role of surface states for both radiative recombination and the first-step TET. Stronger intrinsic NIR emission of AgBiS_2_ NCs also leads to a stronger TTA-UC when combined with TCA and rubrene (Figure S9). On the other hand, when more nonradiative recombination channels are present, as in the room and high temperatures, significant drops of TTA-UC efficiency would be observed. To test this, we performed low-temperature NIR-to-visible photon UC measurements of the AgBiS_2_-TCA-rubrene system. Figure S10a shows that as the temperature decreases, the UCQY efficiency increases. The increase of UCQY at low temperatures can be ascribed to the reduced nonradiative loss channels, which lead to an enhanced first step TET, and the extended triplet lifetime of TCA, which results in an enhanced second step TET. However, the UCQY also depends on the rate of diffusion of annihilator molecules and frequency of TCA-rubrene and rubrene–rubrene collision.^32^ At low temperature, the decreased rate of diffusion and random collision would lead to a reduction of UCQY. Due to these two competing factors, we obtained the highest UC emission intensity at 200 K, with a UCQY of 17% (normalized to 100%) (Figure S10d). This result suggests a complicated interplay between these temperature-dependent factors, and the exact mechanism requires further investigation (Note S4).

### Triplet Sensitization Mechanism

**Figure 3:**
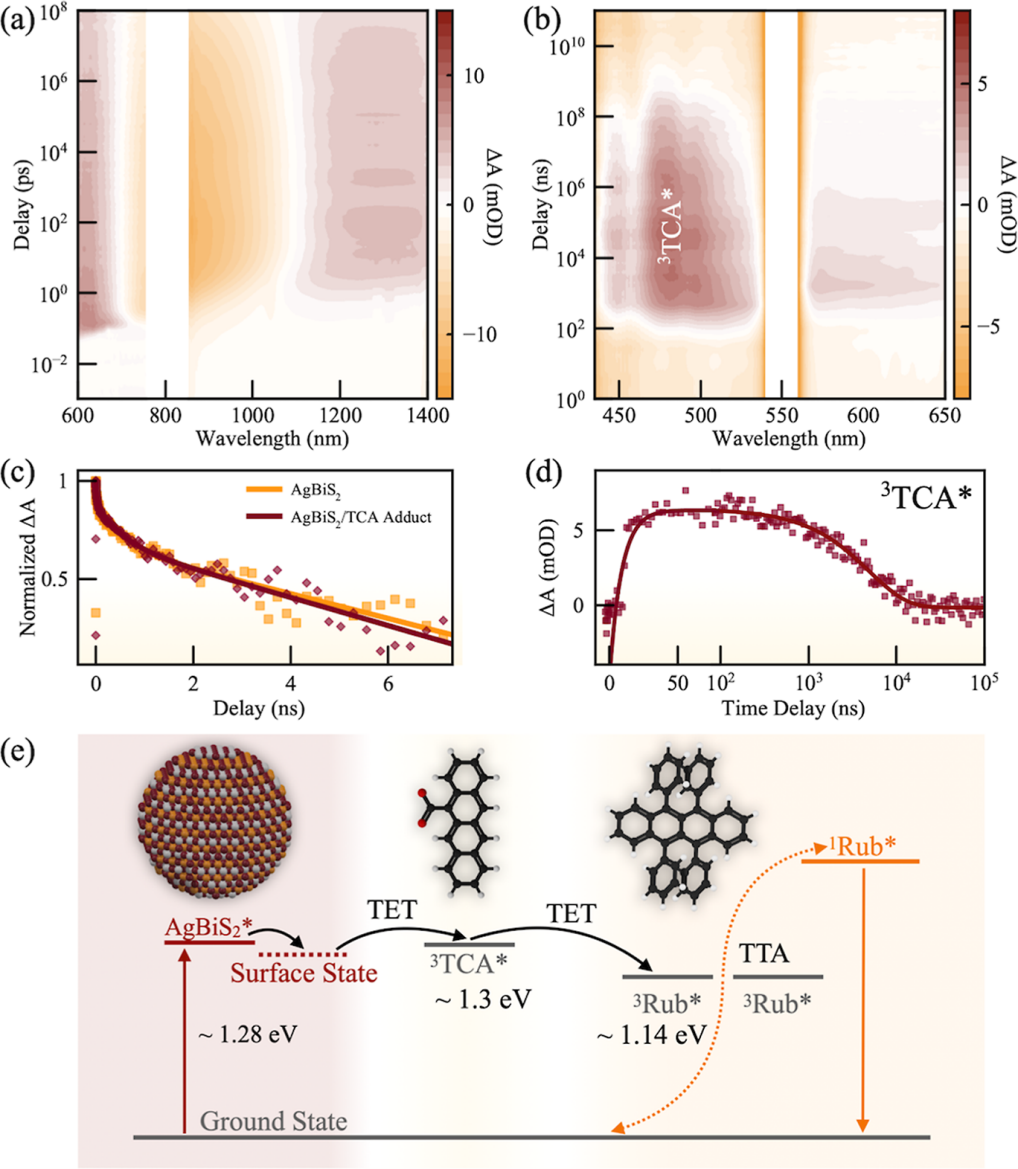
2D pseudocolor picosecond TA spectrum of AgBiS_2_ NCs (a) in hexane excited at 800 nm and nanosecond TA spectrum of AgBiS_2_/TCA (b) in toluene excited at 550 nm. (c) Kinetics of the GSB signal at 900 nm of AgBiS_2_ NCs (orange) and AgBiS_2_/TCA (Red) with fittings (solid lines) excited with 800 nm. (d) Kinetics of the ^3^TCA* PIA signal at 485 nm of AgBiS_2_/TCA with fitting (solid line) excited with 550 nm. (e) Schematics of the TTA-UC system comprising the AgBiS_2_ NC sensitizer, TCA acceptor, and rubrene annihilator.

To further elucidate the TET mechanism between AgBiS_2_ NCs and TCA, pump–probe TA spectroscopy was performed on AgBiS_2_ and AgBiS_2_-TCA solutions. By adjusting the pump–probe delays, we can probe the ultrafast energy transfer dynamics at the NC-TCA interface at picosecond-nanosecond range and probe the dynamics of long-lived triplet excited states at the nanosecond-microsecond range. The pump wavelength is selected as 800 nm, which selectively excites AgBiS_2_ NCs but not TCA molecules, and both visible and NIR broad bands were probed. Figure 3a shows the evolution of the TA signal for the AgBiS_2_ NCs. A broad ground state bleaching (GSB) negative signal centered at around 960 nm was observed in all samples, corresponding to the indirect band gap of AgBiS_2_ NCs (Figure S11).^33^ Simultaneously, a photoinduced absorption (PIA) positive signal can be observed at 1100–1400 nm.

To better observe the triplet features from TCA, we further used a 550 nm pump to excite the AgBiS_2_-TCA solutions, which only excite the NC and not TCA molecules. Figure 3b shows that the AgBiS_2_/TCA adduct exhibited an additional PIA signal in the range of 440–520 nm, which can be assigned to the T_1_ → T_*n*_ absorption of TCA triplets (^3^TCA*). For precise determination of TET dynamics, the GSBs of both AgBiS_2_ NCs and the AgBiS_2_/TCA adduct as well as the ^3^TCA* PIA signal were fitted with exponential functions. Figure 3c shows the kinetics of the GSB signal of AgBiS_2_ NC and AgBiS_2_/TCA. Although the PL lifetime was significantly shortened compared to pristine AgBiS_2_ NCs, the GSB decay dynamics exhibited a different behavior. Specifically, the GSB signal of AgBiS_2_/TCA adducts decayed only slightly faster than that of pristine AgBiS_2_ NCs within the first 6 ns, despite the effective quenching of PL. This suggests that the TET process is outcompeted by the hole-trapping process within this time frame. The decay of GSB signals was not the result of recombination, rather, the depopulation of band edge holes to a trap state. Similar carrier dynamics had also been reported on the CuInS_2_ system.^27^ Notably, the rise of ^3^TCA* PIA was observed only after the GSB signal disappeared (∼10.7 ns, Figure 3d), implying that trapped excitons are capable of sensitizing the TCA triplet state. The proposed surface-state mediated TET mechanism is in agreement with our analysis for the PL mechanism, where the PL of AgBiS_2_ NCs originates from the free-to-bound exciton transition. Figure 3d presents the kinetics of the ^3^TCA* signals. Exponential fitting gives an average rise time ^3^TCA* of 10.7 ns, which agrees with the *k*_TET_ calculated from TRPL. The average lifetime of ^3^TCA* was found to be 4.7 μs. Figure 3e presents a schematic illustration for the surface-state mediated TET mechanism sensitized by AgBiS_2_ NCs and we quantified the quantum yields of each elementary step in TTA-UC (Note S5).

## Discussion

Our work thus presents the first observation of radiative NIR emission and triplet sensitization TTA-UC from AgBiS_2_ NCs. To confirm the proposed mechanism, which is based on the free electron and trapped hole recombination, we need to carefully exclude other possible origins. For instance, it is possible that some binary impurities (Bi_2_S_3_ or Ag_2_S) were formed in small quantities during the synthesis of ternary NCs. We exclude the formation of Bi_2_S_3_ NCs since it has never been reported to display any luminescence. In addition, our synthetic condition with an excess of silver will favor the formation of AgBiS_2_ instead of Bi_2_S_3_. Ag_2_S NCs, on the other hand, were reported to exhibit NIR emissions under certain synthetic conditions. It is worth noting that a great excess of silver content (feed ratio of Ag/S > 1.4) indeed results in the formation of Ag_2_S NCs, which was confirmed by PXRD (Figure S2) and ICP-OES elemental analysis (Table S1). However, these Ag_2_S NCs were found to be nonluminescent. Only when the feed ratio of Ag/S is in the range of 1.1–1.3, without any detectable Ag_2_S NCs, we were able to observe the strong NIR emission. Additionally, when Ag_2_S NCs were intentionally synthesized, they cannot photosensitize TCA molecules or perform the NIR-to-visible TTA-UC (Figure S12). Therefore, we exclude the impurity phases as the possible origin and conclude that the radiative NIR emission and triplet sensitization is indeed related with the off-stoichiometric AgBiS_2_ NCs.

**Figure 4:**
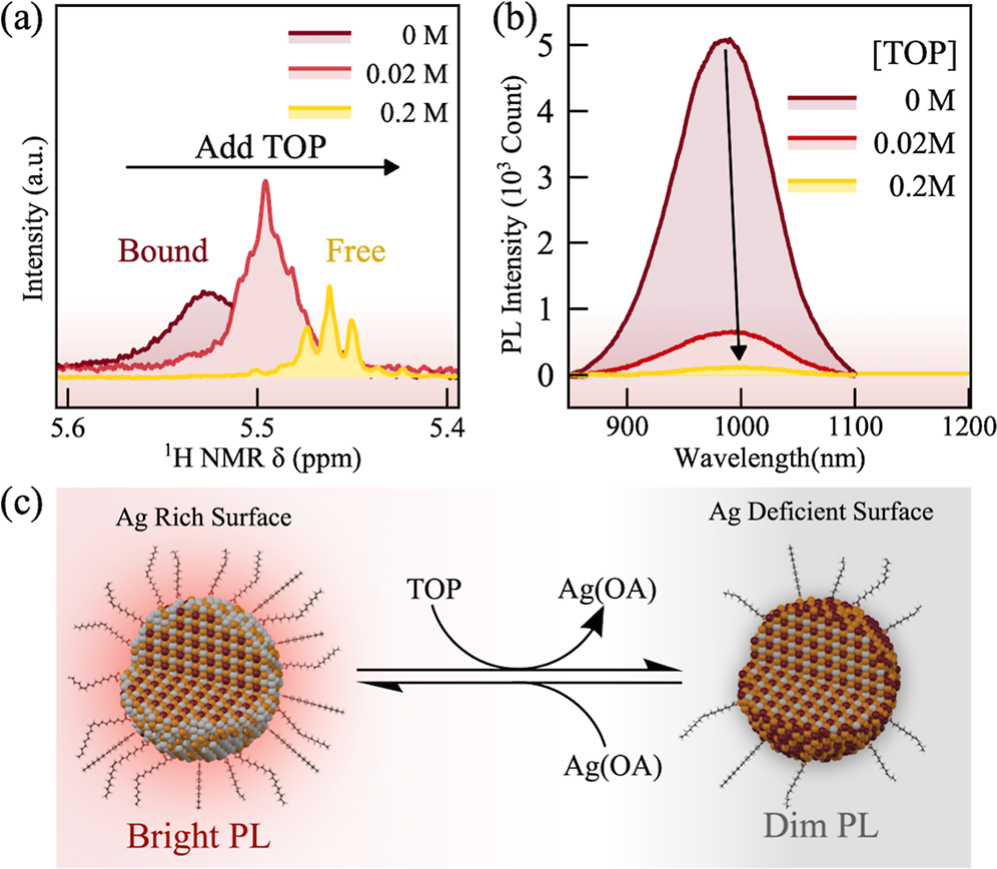
(a) Vinyl region of the ^1^H NMR spectrum of OA-capped AgBiS_2_ NCs shows displacement of Ag oleate on treatment with increasing concentration of TOP. (b) Normalized PL spectra of AgBiS_2_ NCs treated with different concentrations of TOP. (c) Schematic depiction of Z-type ligand displacement promoted by addition of Lewis base leading to PL intensity quenching and treating silver deficient AgBiS_2_ NCs with Ag oleate leading to PL intensity enhancement.

To understand the origin of NIR PL with the silver-rich AgBiS_2_ NCs, we systematically modulated the surface silver stoichiometry by ligand exchange reactions. Since the reaction mixture possesses excess silver ions and OA ligands, the surface of the as-synthesized AgBiS_2_ NCs is likely passivated by X-type (deprotonated oleate), L-type (OA), and Z-type [silver oleate, Ag(OA)] ligands. This is confirmed by the solution ^1^H NMR spectroscopy (Figure S13), where broaden peaks corresponding to OA ligands were observed in the as-synthesized AgBiS_2_ NCs. Since the emissive AgBiS_2_ NCs show a silver-rich stoichiometry, we envision that excess silver ions are present on the surface as the Z-type ligand, that is, silver oleate Ag(OA). These neutral Z-type ligands can be readily replaced by other Lewis bases such as trioctylphosphine (TOP).^34^ As shown by Figure 4a, titration of TOP to AgBiS_2_ NCs results in the removal of Ag(OA), as indicated by the sharpening of the vinyl peak and the shift to lower ppm in NMR spectrum. Interestingly, we found that the removal of Ag(OA) leads to a significant quenching of the NIR PL (Figure 4b), suggesting the critical role of Ag(OA) ligands for NIR emission. Similar PL quenching was also observed in AgBiS_2_ NCs when adding other Lewis bases (Figures S14 and S15). On the other hand, treating silver-deficient AgBiS_2_ NCs (feed ratio: 1.1:1:1) with silver oleate leads to an enhancement of PL intensity by around 1.25 times. Further addition of silver oleate reduces the PL intensity as it converts NCs to the nonemissive Ag_2_S phase (Figure S16). Therefore, our control experiments indicate that surface silver oleate ligands, which serve as the hold scavenger, are the key for switching on the NIR emission in AgBiS_2_ NCs. The conversion between bright and dim states of AgBiS_2_ NCs is summarized as a schematic in Figure 4c. On one hand, excess silver oleate can act as surface hole traps, similar to Cu^+^ in CuInS_2_ NCs, providing the radiative recombination center for free electrons. This is the essential step to circumvent the indirect bandgap nature of AgBiS_2_ NCs. The surface silver oleate can also act as a Z-type ligand and passivate uncoordinated sulfur sites, suppressing nonradiative recombination.^34^ On the other hand, these surface-trapped excitons in AgBiS_2_ NCs can further transfer their energy to a molecular triplet acceptor, enabling the efficient TET and TTA-UC processes.

## Conclusions

In conclusion, we demonstrated, for the first time, that an off-stoichiometric silver-rich AgBiS_2_ NCs can switch on the radiative NIR emission. A mechanistic study shows that excess silver ions are located on the surface as silver oleate, which serves as a hole trap. The NIR emission origins from the recombination of a photogenerated electron and a trapped hole located on silver oleate. This radiative surface state can serve as an important intermediate to sensitize molecular triplets of TCA molecules with an efficiency of 90.8%. When combined with rubrene molecules, our silver-rich AgBiS_2_ NCs can achieve TTA-UC with a UCQY of 10.5% (normalized to 100%) at room temperature, which can be further improved to ∼17% at 200 K. Our work demonstrates a new strategy to modulate the photophysical properties of I–V–VI_2_ ternary NCs that were previously unattainable. Our silver-rich AgBiS_2_ NCs represent a new easy-to-fabricate, nontoxic, NIR emitter and efficient molecular triplet sensitizer.

## Methods

### Chemicals

OA (85%), propylamine (99%), dibutylamine (99%), trioctylamine (99%), pentanol (99%), and tetrahydrofuran (99%) were purchased from Aladdin. Bismuth acetate [Bi(OAc)_3_, 99.5%], silver acetate [Ag(OAc), 99%], and indocyanine green (95%) were purchased from Macklin. HMS (98%) and TOP (90%) were purchased from Energy Chemical. Rubrene (99%) was purchased from Bidepharm. TCA was synthesized according to a literature method.^35^ Dimethyl sulfoxide (DMSO), anhydrous hexane, anhydrous toluene, acetone, and 1-octadecene (ODE, 90%), nitric acid were purchased from Sigma-Aldrich. ICP Calibration standard 23 analytes, 1000 μg/mL in water with dil. nitric acid, were purchased from Agilent.

### Synthesis of AgBiS_2_ NCs

AgBiS_2_ NCs were synthesized according to literature methods^9^ with modifications. In a typical synthesis, 1 mmol of Bi(OAc)_3_, 6 mL OA, and 4.5 mL of ODE were loaded into a 50 mL three-necked flask, followed by degassing under vacuum at room temperature for 30 min under continuous stirring. The temperature was then increased to 105 °C while still under vacuum and maintained for 1 h to remove all oxygen and moisture. After 1 h, the flask was refilled with nitrogen. 1.2 mmol Ag(OAc) powder was quickly added to the solution under vigorous stirring, and allowed to completely dissolve while maintaining 105 °C. To prepare sulfur precursors, 1 mmol of HMS was dissolved in 0.5 mL of oxygen-free and moisture-free ODE in a N_2_-filled glovebox. After Ag(OAc) powder was completely dissolved, the heating was turned off while the flask is still in the heating mantle to slowly cool down. The HMS/ODE sulfur precursor is quickly injected into the reaction mixture once its temperature reaches 100 °C. Upon injection, the temperature of the reaction mixture will spike up to 102 °C and immediately turn black. The reaction was then allowed to cool down slowly while being in the mantle for 45 min.

The NC is purified via size-selective precipitation (Figure S17). In general, size-selective precipitation yields the same crystalline phase (Figure S18) of AgBiS_2_ with different particle sizes. Briefly, the crude NC solution is diluted with 4 mL of hexane and split evenly into 2 centrifuge tubes. 13 mL of anhydrous acetone is added to each tube and centrifuged at 11,000 rpm for 3 min. The supernatant is kept and the remaining solid is discarded. To the remaining supernatant, 20 mL of anhydrous acetone is added and centrifuged at 11,000 rpm for 5 min. The supernatant is discarded, while the remaining precipitate is redispersed is 10 mL of anhydrous hexane. The solution is then filtered with a 0.22 μm Nylon membrane syringe filter. NCs of other Ag/Bi:S ratios were synthesized by changing the amount of Ag(OAc) powder added or the volume of HMS/ODE precursor injected, while keeping the other conditions unchanged.

It is worth noting that during the synthesis, the time between the addition of Ag(OAc) powder and the injection of HMS/ODE should not be longer than 30 min to prevent the formation of unwanted Ag NCs. The color of the reaction mixture should remain transparent or off-yellow before the injection of HMS/ODE. Browning or blackening of reaction mixture prior to HMS/ODE injection suggests the formation of large amount of Ag NCs, which could deviate the Ag/Bi feeding ratio from the intended amount.

### Preparation of AgBiS_2_-TCA Adduct

In a N_2_-filled glovebox, TCA Powder was added to 2 mL of AgBiS_2_ hexane solution in a glass vial. The vial was then sonicated for 30 s. The mixture was then filtered twice using a 0.22 μm nylon membrane syringe filter to obtain a clear solution containing the AgBiS_2_-TCA adduct.

### Preparation of the UC Mixture

The AgBiS_2_-TCA hexane solution is dried in vacuo. In a N_2_-filled glovebox, 2 mL of 20 mM rubrene solution is added to the dried powder to make a 25 μM NC solution. The mixture is stirred vigorously to ensure complete dispersion. The exact NC loading is confirmed via UV–vis absorption spectroscopy afterward in a 1 mm path length cuvette.

### Ag Oleate Postsynthetic Surface Passivation

To synthesize silver oleate, 3 mmol of Ag(OAc) and 6 mL of OA were added to a 50 mL three-necked flask and pumped under vacuum with vigorous stirring at room temperature for 30 min to remove oxygen. The reaction mixture was then heated to 105 °C for 30 min or until the silver acetate fully dissolved, forming a clear solution. The mixture was removed from heat, backfilled with nitrogen (N_2_), and allowed to cool to room temperature. The resulting white waxy mixture was washed three times with acetone under suction filtration to remove excess OA, yielding a white solid—silver oleate. This silver oleate was then dried under vacuum for 24 h and stored in a glovebox protected from light. To prepare a silver oleate/toluene solution, 15 mg of silver oleate was added to 15 mL of anhydrous toluene inside a glovebox and sonicated for 30 min to homogenize. This solution was then incrementally added at 10 μL intervals to a 1 mL AgBiS_2_/hexane solution (OD@808 nm = 0.35) with vigorous stirring, and the PL spectrum was measured 5 min after each addition.

### TTA-UC Measurements

The AgBiS_2_/TCA/rubrene UC mixture was sealed in a 1 cm path length airtight cuvette for the TTA-UC measurements. The sample was excited by an 808 nm *cw*-laser while being stirred vigorously during measurement. The laser beam was focused onto the sample using a quartz lens with a beam diameter of ∼0.20 mm measured using the knife edge method (Note S6). A lens system was used to collect the fluorescence, which was fiber-coupled to a spectrometer. The normalized TTA-UC quantum yield (η_UC_) was calculated according to the following equation
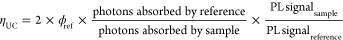
where φ_ref_ is the PLQY of the reference (rubrene: PLQY = 98% for 532 nm excitation). The number of photons absorbed by the sample or reference per second was calculated from their absorbance, excitation power, and excitation photon energy.

## PLQY Measurements

AgBiS_2_ NCs dispersed in anhydrous hexane were sealed in a 1 cm path length airtight cuvette for PLQY measurements. The sample was excited by an 808 nm in the same setup as the TTA-UC measurements. The PLQY Φ_PL_ was calculated according to the following equation
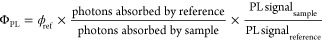
where φ_ref_ is the PLQY of the reference (Indocyanine Green: PLQY = 10.6% for 678 nm excitation in DMSO).^36^ The number of photons absorbed by the sample or reference per second was calculated from their absorbance, excitation power, and excitation photon energy.

### Temperature-Dependent TTA-UC Measurement

The AgBiS_2_/TCA/Rubrene UC mixture was loaded and sealed in a 1 mm path length airtight quartz cell using UV-set resin. The cell was then placed into a cooling chamber cooled with liquid nitrogen. The temperature of the chamber, monitored by a thermocouple, was gradually increased using a heating element. UC emission spectra were measured 5 min after reaching each target temperature to ensure complete thermal equilibrium.

### Characterization of NCs

TEM images were obtained using a JEOL 2010F field emission electron microscope. The NC samples were drop-casted onto an ultrathin carbon film supported by a Cu grid. PXRD patterns were obtained using a Rigaku Miniflex with Cu Kα radiation. The scanning rate was set as 1° per min.

To prepare analyst solution for elemental analysis, AgBiS_2_ NC samples prepared according to the procedures above were further washed twice with acetone to remove organic ligands. 5 mg of washed and dried sample pellets are then digested with 15 mL of 1.5 M nitric acid at 80 °C overnight to ensure no solid material remains. The digested analyze solution was then filtered using a 0.22 μm nylon filter and stored in a tightly capped centrifuge tube. Calibration solutions were prepared by diluting commercial multielement calibration standard diluted with 1.5 M nitric acid. Elemental atomic ratio was obtained using a PerkinElmer’s Avio 200 hybrid-scanning ICP-OES.

### TA Spectroscopy

The femtosecond pump–probe TA measurements^37^ were performed using a regenerative amplified titanium/sapphire laser system (800 nm, 70 fs, 6 mJ per pulse and 1 kHz repetition rate; Coherent) as the laser source and an ultrafast spectrometer. Briefly, the 800 nm output pulse from the regenerative amplifier was split into multiple parts.

A portion of the excitation beam was used directly to excite the AgBiS_2_ NCs, while less than 10% of the beam was attenuated using a neutral-density filter and focused into a nonlinear crystal to generate a white-light continuum, which served as the probe beam. The probe beam was directed onto the sample using an aluminum parabolic reflector, after which it was collimated and subsequently focused into a fiber-coupled spectrometer equipped with complementary metal–oxide–semiconductor sensors for detection at a frequency of 1 kHz.

The intensity of the pump pulses employed in the experiment was modulated using a variable neutral-density filter wheel, and the temporal delay between the pump and probe pulses was precisely controlled via a motorized delay stage. To facilitate pump–probe measurements, the pump pulses were modulated at 500 Hz using a synchronized optical chopper.

Nanosecond TA spectroscopy was performed using an EOS spectrometer (Ultrafast Systems). The pump beam was generated in the same way as for the femtosecond TA experiments described above.

A different white-light continuum (380–1700 nm, 0.5 ns pulse width and 20 kHz repetition rate) was used, which was generated by focusing a Nd/YAG laser into a photonic crystal fiber. The delay time between the pump and probe beams was controlled using a digital delay generator (CNT-90, Pendulum Instruments).

The samples were placed in 1 mm airtight cuvettes in a glovebox and measured under ambient conditions.

### Time-Resolved PL Decay Spectroscopy

The TRPL decay was measured using a homemade time-correlated single-photon-counting setup. The excitation source was a supercontinuum laser with appropriate optical filters. The PL photons were collected with a lens, filtered, and then detected using an avalanche photodiode detector.

## Supporting Information Available

Additional structural and morphology characterization of NCs, steady-state (absorption, PL) and time-resolved (TRPL) spectroscopic data, NMR data, and power-dependent, temperature-dependent TTA-UC data and analysis; and discussion on the merit of using AgBiS_2_ NCs as the NIR triplet sensitizer for TTA-UC, the accuracy of relative methods, the factors that contribute to UCQY under 980 nm excitation, and at various temperatures (PDF)
