# Supplementary material for: Triplet Sensitization Photon Upconversion Using Near-Infrared Indirect-Bandgap AgBiS2 Nanocrystals
Source: J Am Chem Soc. 2025 Apr 9;147(16):14015–23. doi: 10.1021/jacs.5c04015 (PMC12023030; doi:10.1021/jacs.5c04015)
Supplement: Supplementary file 1 — ja5c04015_si_001.pdf [file ja5c04015_si_001.pdf]

Supplementary Information for

Triplet Sensitization Photon Upconversion Using Near-Infrared Indirect-Bandgap $AgBiS_2$ Nanocrystals

Kin Ting Chang,[1#] Wenfei Liang,[1#] Shaokuan Gong,[2] Pang Ho Yeung, [1] Jianning Feng, [1] Xihan Chen,[2] Haipeng Lu[1*]

[1] Department of Chemistry, The Hong Kong University of Science and Technology, Clear Water Bay, Kowloon, Hong Kong, China (SAR)
[2] Department of Mechanical and Energy Engineering, Southern University of Science and Technology, Shenzhen, Guangdong 518055, China
[#] These authors contributed equally: Kin Ting Chang and Wenfei Liang

Corresponding Author
Haipeng Lu[*]: haipenglu@ust.hk

Content

Figure S1. Color map of $AgBiS_2$ NCs synthesis feed ratio and their PL intensity	P3
Figure S2. Powder XRD pattern for NC samples synthesized under different feed ratio and structure of $AgBiS_2$ with lattice plane	P3
Figure S3. PL spectra of $AgBiS_2$ NCs at different excitation powers	P4
Figure S4. Time-resolved PL decay of Ag-rich $AgBiS_2$ NCs	P4
Figure S5. Dependence of PL intensity on their Ag:Bi ratio measured from ICP-OES.	P5
Figure S6. Absorption spectra of TCA molecules attached on the surface of $AgBiS_2$ NCs and free TCA molecules	P5
Figure S7. Calibration curve for the molar extinction coefficient of $AgBiS_2$ NCs.	P6
Figure S8. TTA-UC Emission Spectra of $AgBiS_2$ /TCA Sensitized system excited by 980 nm *cw*-laser	P6
Figure S9. Dependence of upconversion emission on varying feed ratio and dependence of UC intensity on the PL intensity of NCs	P7
Figure S10. UC emission spectra, intensity and UCQY at low temperature	P8
Figure S11. Indirect band gap tauc plot analysis of $AgBiS_2$ NCs	P9

Figure S12. Absorption and PL spectra of Ag_2S NCs and Ag_2S/TCA adduct	P10
Figure S13. ^{1}H NMR spectrum of oleic acid capped $AgBiS_2$ NCs and free oleic acid	P11
Figure S14. Vinyl Region of the ^{1}H NMR spectra of $AgBiS_2$ NCs treated with different Lewis bases	P12
Figure S15. PL spectra of $AgBiS_2$ NCs treated with different Lewis bases	P12
Figure S16. PL spectra and integrated PL intensity of $AgBiS_2$ NCs treated with different concentration of silver oleate	P13
Figure S17. Size distribution histograms, TEM images, absorption and PL spectra of $AgBiS_2$ NCs obtained from different stages of size selective precipitation	P14
Figure S18. PXRD patterns of $AgBiS_2$ NCs obtained from different stages of size selective precipitation	P14
Table S1. ICP-OES Elemental analysis of $AgBiS_2$ NC samples synthesized from different feeding ratio	P15
Table S2. Fitting parameters for the TRPL kinetics for $AgBiS_2$ NCs and $AgBiS_2$/TCA adduct.	P15
Supplementary Note S1. Significance of $AgBiS_2$ NCs as TTA-UC sensitizers	P16
Figure S19. Absorption coefficient of different common photovoltaic materials	P17
Supplementary Note S2. TET-1 rate and efficiency estimation from TRPL kinetics	P17
Supplementary Note S3. Lower UCQY for 980 nm excited upconversion	P18
Supplementary Note S4. Enhanced UCQY at low temperature	P18
Supplementary Note S5. Efficiency for each step in the upconversion process	P19
Supplementary Note S6. Reliability for the relative method for UCQY measurement	P20
Figure S20. Beam size determination for 405 nm and 808 nm cw-laser	P20

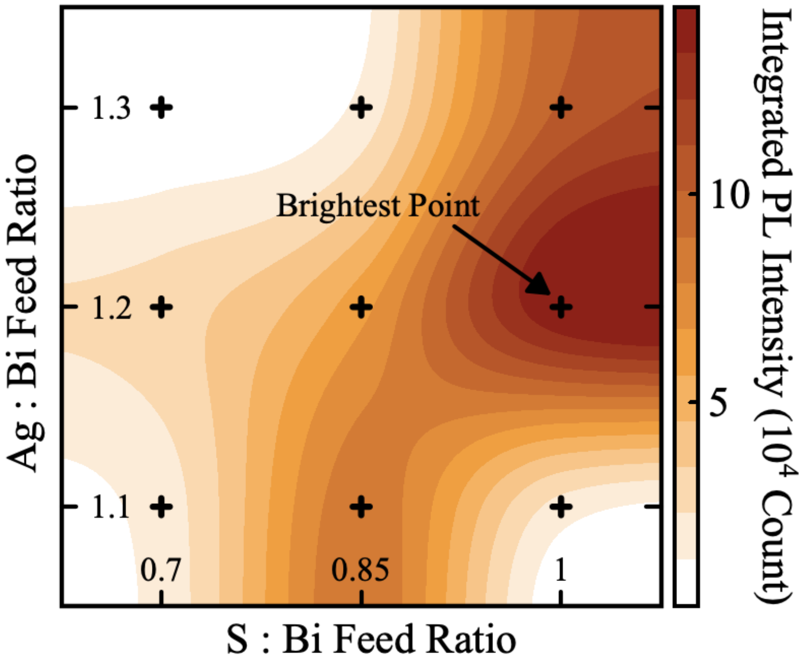

Figure S1. 2D pseudo-colored interpolated map of $AgBiS_2$ synthesis feed ratio variance and their corresponding PL intensity, black crosses indicate the feed ratio that has been synthesized.

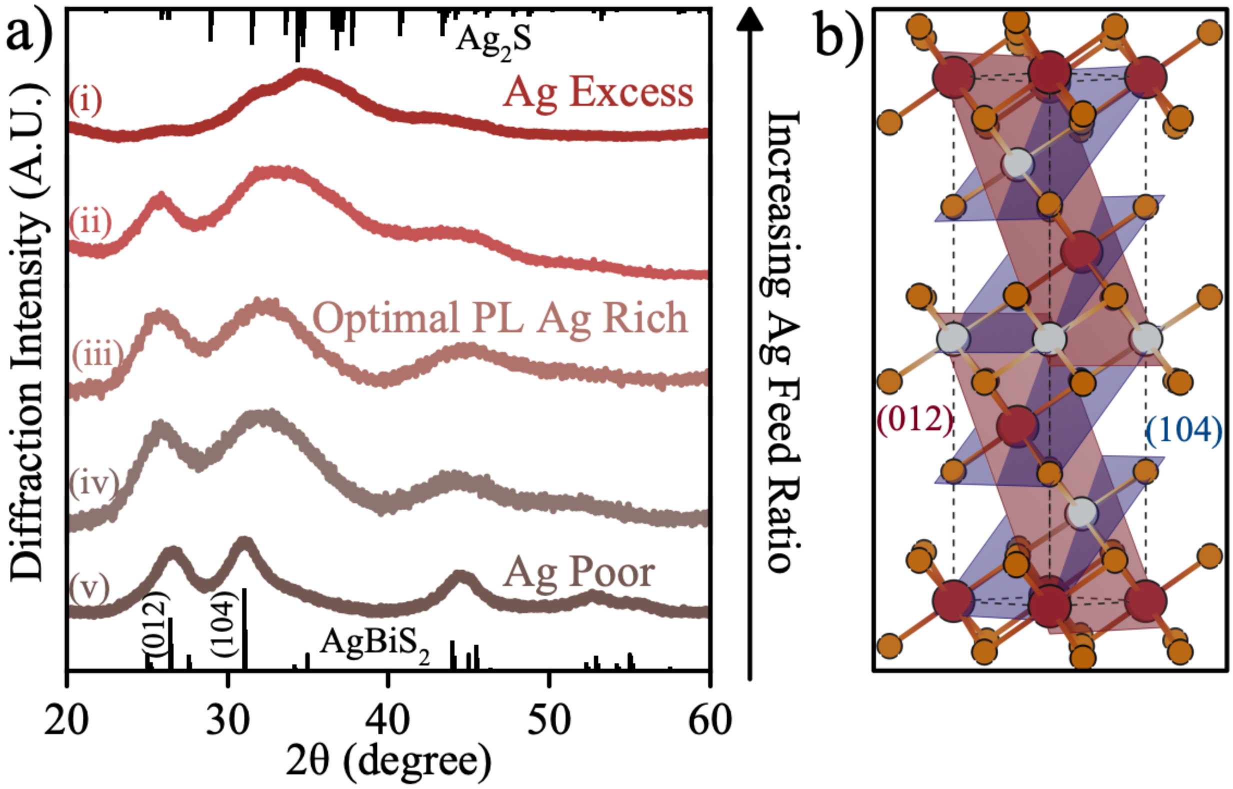

Figure S2. (a) Powder XRD pattern for NC samples synthesized under varying Ag feed ratio, diffraction pattern reference for the matildite phase of $AgBiS_2$ (ICDD 96-901-1025) (bottom), and the Acanthite phase of Ag_2 S (ICDD 96-900-0254) (top). The nominal Ag:Bi:S feed ratios are (i) 1.3:1:0.85, (ii) 1.3:1:1, (iii) 1.2:1:1, (iv) 1.1:1:0.85, and (v) 0.8:1:1. (b) Structure of the matildite phase of $AgBiS_2$, colored planes represent the lattice planes (012) (red) and (104) (blue).

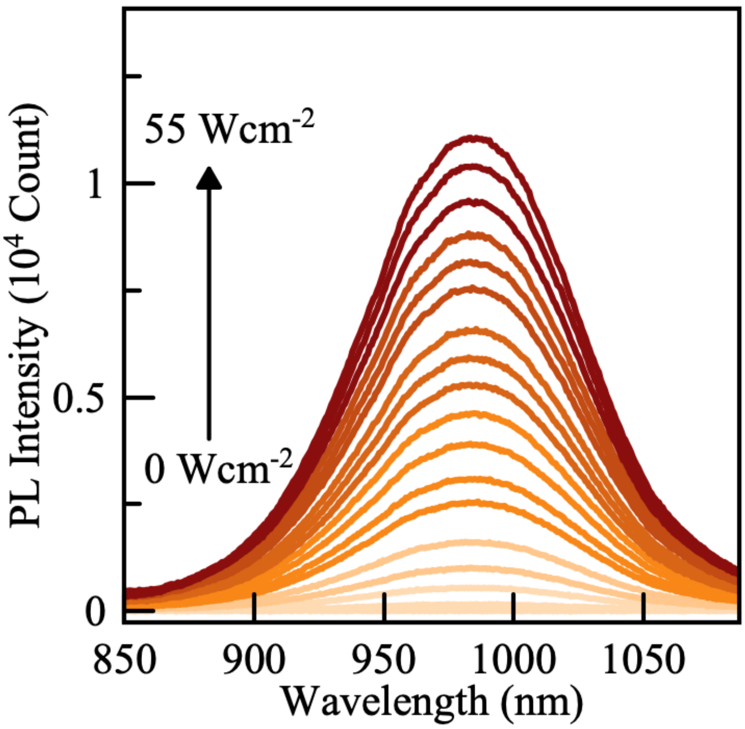

Figure S3. PL spectra of $AgBiS_2$ NCs colloidal solution in anhydrous toluene at different excitation powers (0 – 55 Wcm^{-2}) of 808 nm *cw* laser.

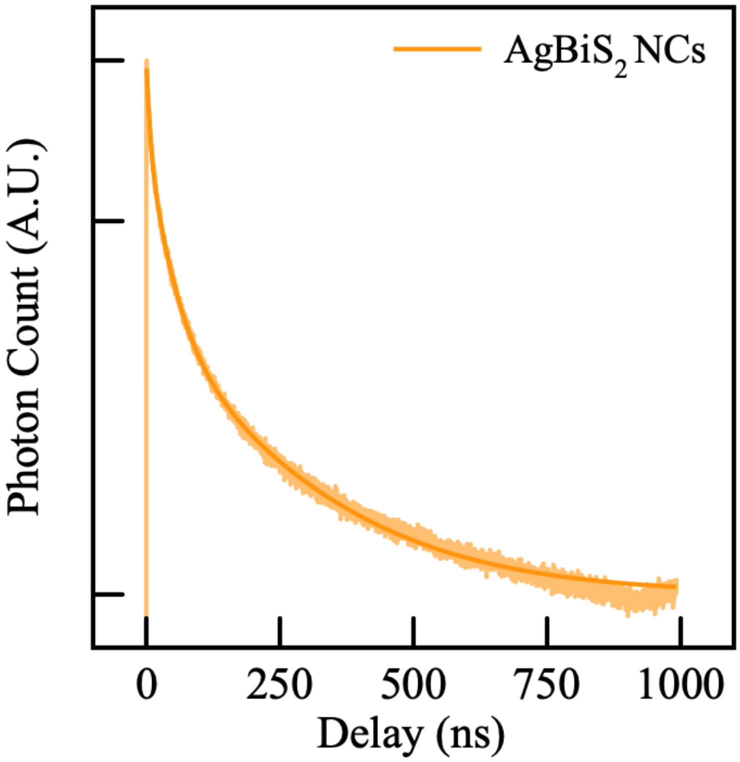

Figure S4. Time-resolved PL decay of Ag-rich $AgBiS_2$ NCs, stretch exponential fitting is indicated by a solid orange line.

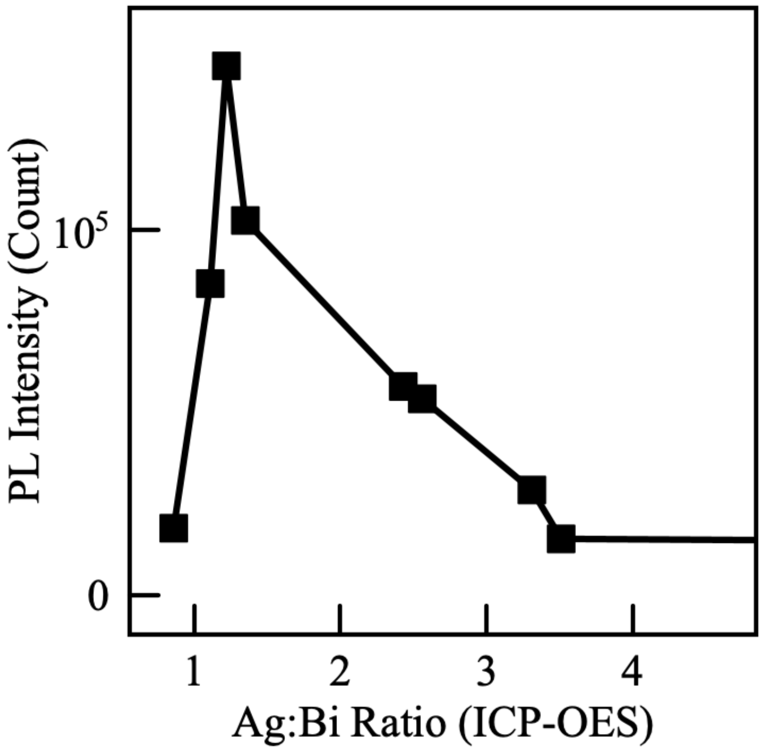

Figure S5. Dependence of integrated PL intensity from different $AgBiS_2$ NCs on their corresponding relative Ag:Bi ratio measured from ICP-OES.

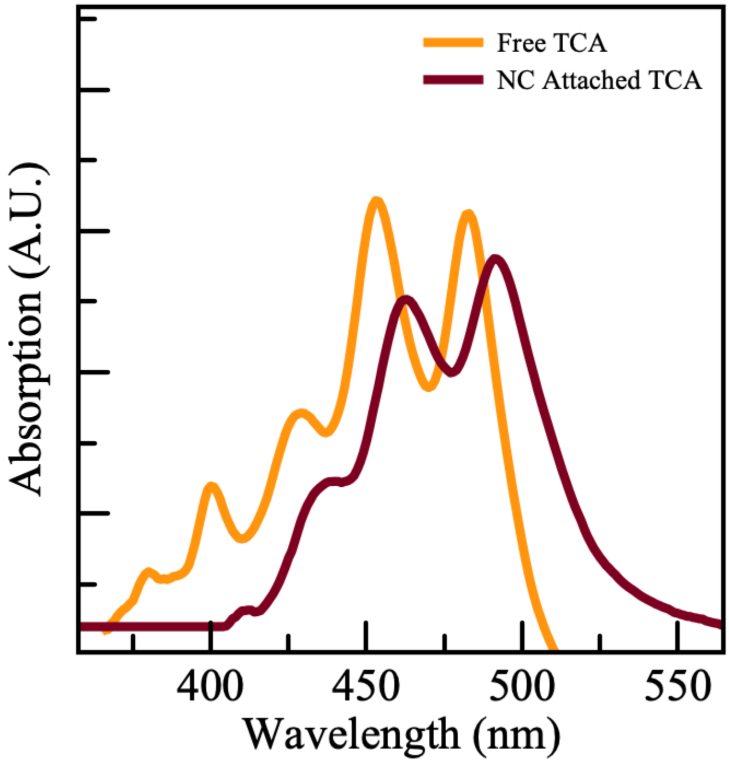

Figure S6. Absorption spectra of TCA molecules attached on the surface of $AgBiS_2$ NCs (red) and free TCA molecules dissolved in toluene (yellow).

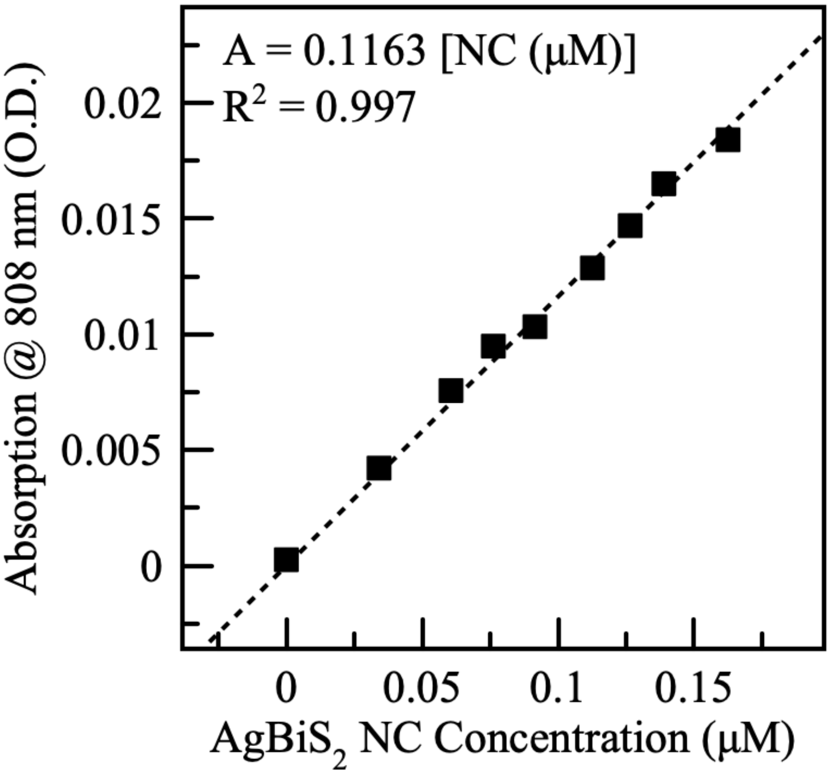

Figure S7. Calibration curve for the molar extinction coefficient at 808 nm of $AgBiS_2$ NCs. The concentrations of $AgBiS_2$ NCs were calculated from ICP-OES results. From linear regression, the molar extinction coefficient of $AgBiS_2$ NCs at 808 nm was determined to be 0.1163 $\mu M^{-1}cm^{-1}$. The absorption coefficient of TCA is 6620 $M^{-1}cm^{-1}$ at 480 nm according to previous literature.[1]

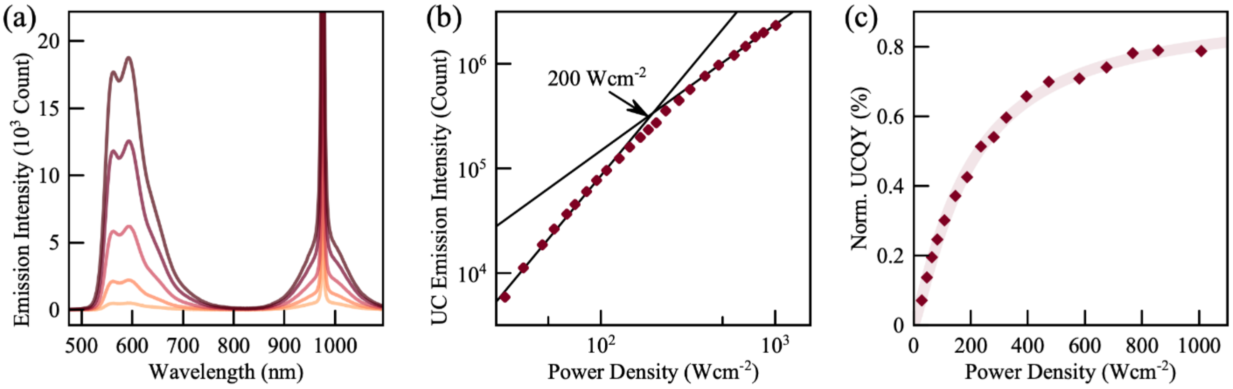

Figure S8. (a) TTA-UC Emission Spectra of $AgBiS_2$/TCA Sensitized system excited by 980 nm *cw*-laser. (b) Dependence of emission intensity on excitation power density. (c) Dependence of normalized UCQY on excitation power. The maximum UCQY achieved is 0.8%

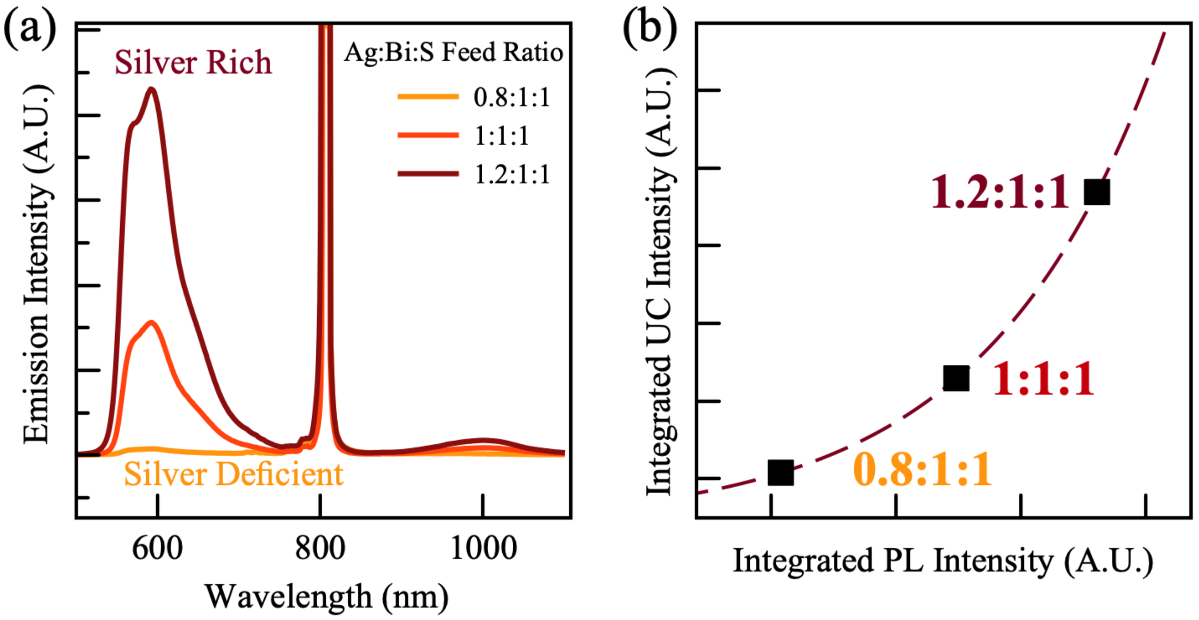

Figure S9. (a) Upconversion emission spectra sensitized by NC samples synthesized under varying Ag:Bi:S feed ratio with the same NC loading. (b) Dependence of integrated UC intensity on the intrinsic PL intensity of NCs synthesized under varying Ag:Bi:S feed ratio, dotted line shows exponential fitting.

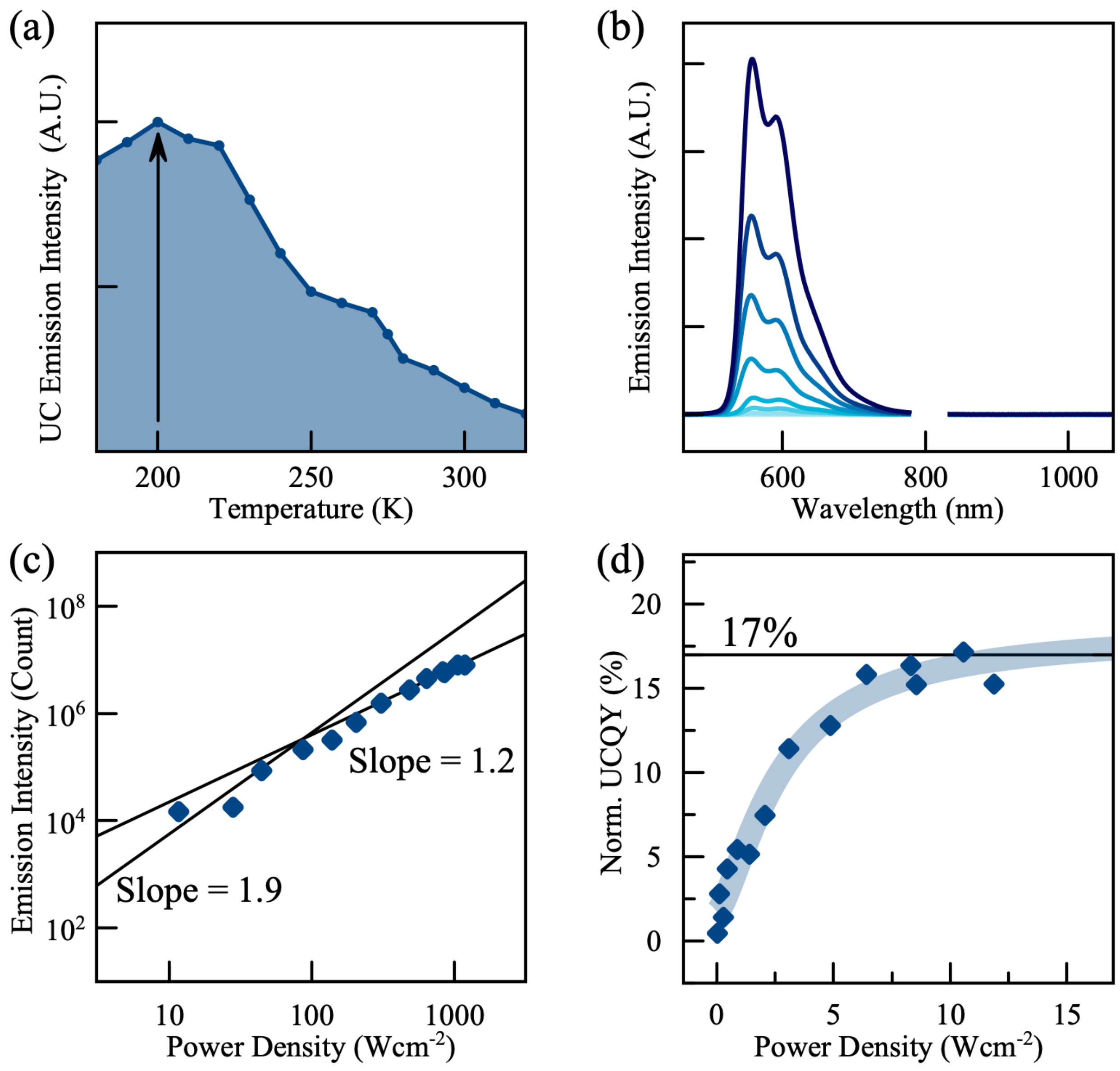

Figure S10. Integrated upconversion emission intensity of $AgBiS_2$ UC mixture in toluene at different temperature from 320K to 180K. Arrow indicates the highest UC emission intensity is attained at 200K (b) Upconversion emission spectra at 200K at different excitation powers of 808 nm laser. (c) UC emission intensity at 200K as a function of excitation power density of the $AgBiS_2$ NC UC system. The crossing point between the low-power quadratic and high-power linear regions is the TTA-UC threshold (d) Dependence of normalized UCQY at 200K on excitation power. The maximum UCQY achieved is indicated in solid line at ~17%.

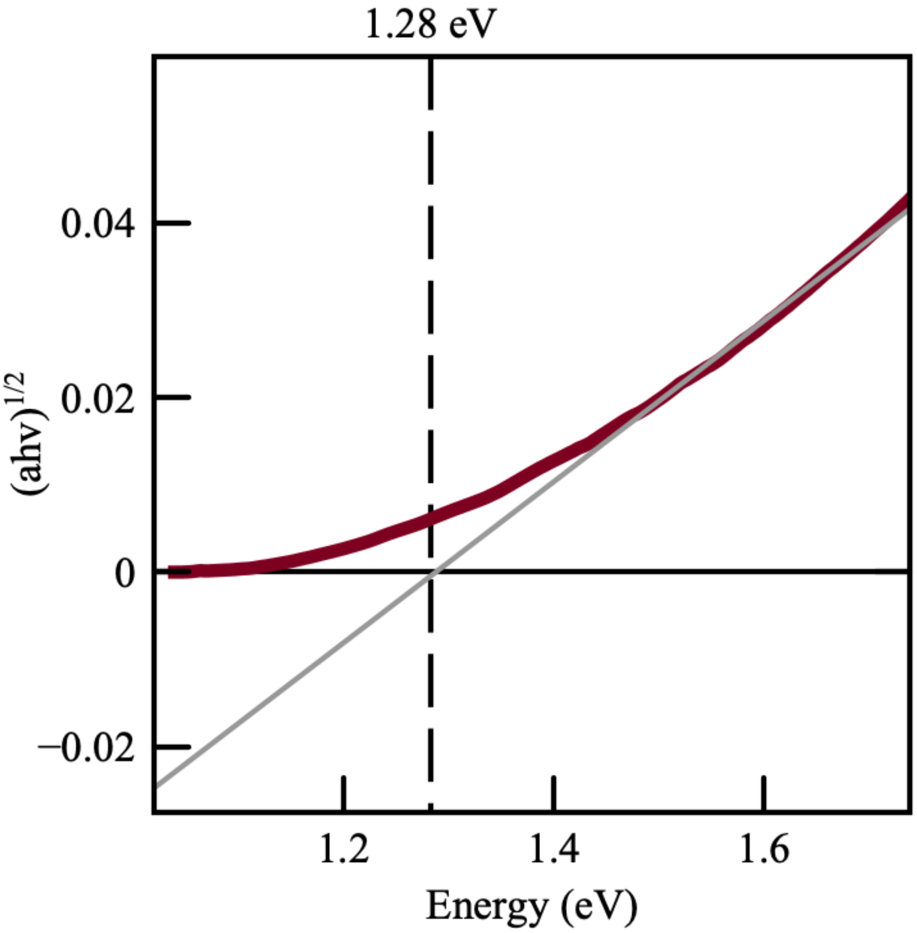

Figure S11. Indirect band gap Tauc plot analysis of the UV-Vis-NIR absorption spectra of $AgBiS_2$ NCs colloidal solution (red). Fitting for the linear region (grey), and the x intercept of the linear fitting (black) indicating the energy of the indirect band gap of the $AgBiS_2$ NCs.

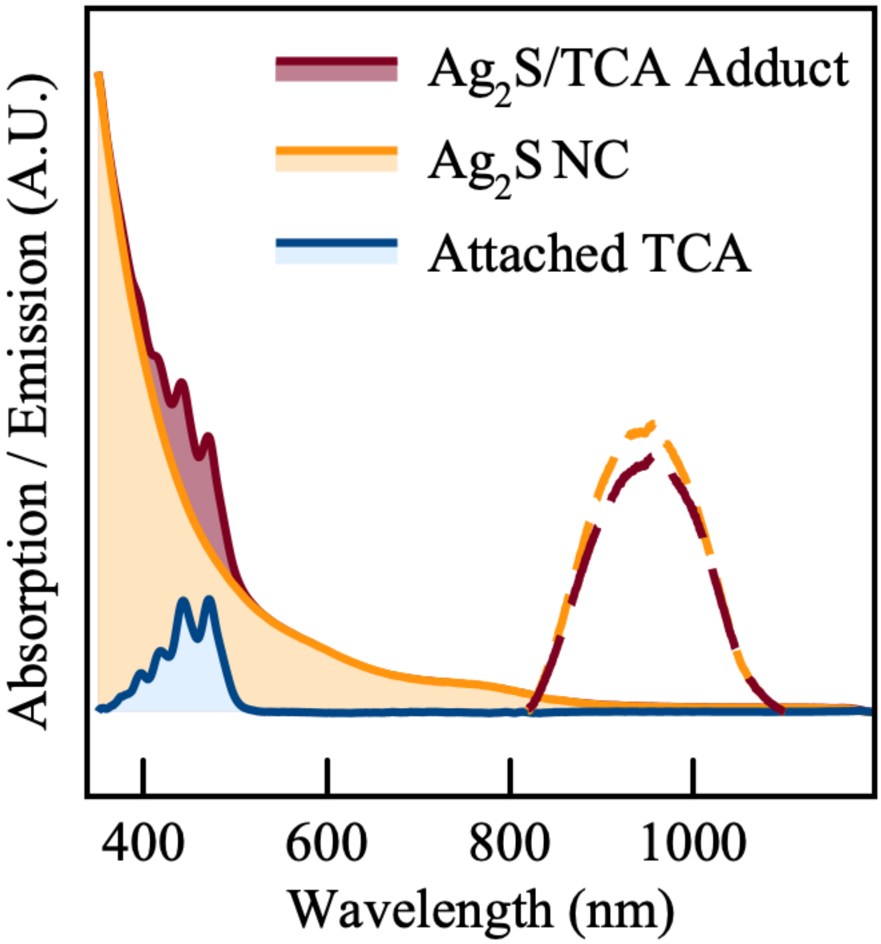

Figure S12. Absorption (solid lines) and PL (dashed lines) spectra of Ag_2 S NCs (Orange) and Ag_2 S/TCA (Red) dispersed in hexane. The absorption spectra of TCA bound to the surface of Ag_2 S NCs (blue) are shown. A comparison of the PL spectra of Ag_2 S NC and the Ag_2 S/TCA adduct indicates that the attachment of TCA molecules does not quench the PL emission from Ag_2 S NCs. Furthermore, when the Ag_2 S/TCA adduct is used to construct a UC system, no upconversion emission is observed.

It is noteworthy that Ag_2 S, unlike $AgBiS_2$, is a well-understood semiconductor, easily synthesized using traditional heat-up and hot-injection methods or even under room temperature. The NC exhibits a distinct NIR emission attributed to its direct band gap. Yet, the literature is devoid of any evidence of Ag_2 S serving as a viable sensitizer or triplet donor for TTA-UC, despite its apparent suitability. This, to some extent, corroborates our findings that Ag_2 S NCs are incapable of sensitizing TTA-UC.

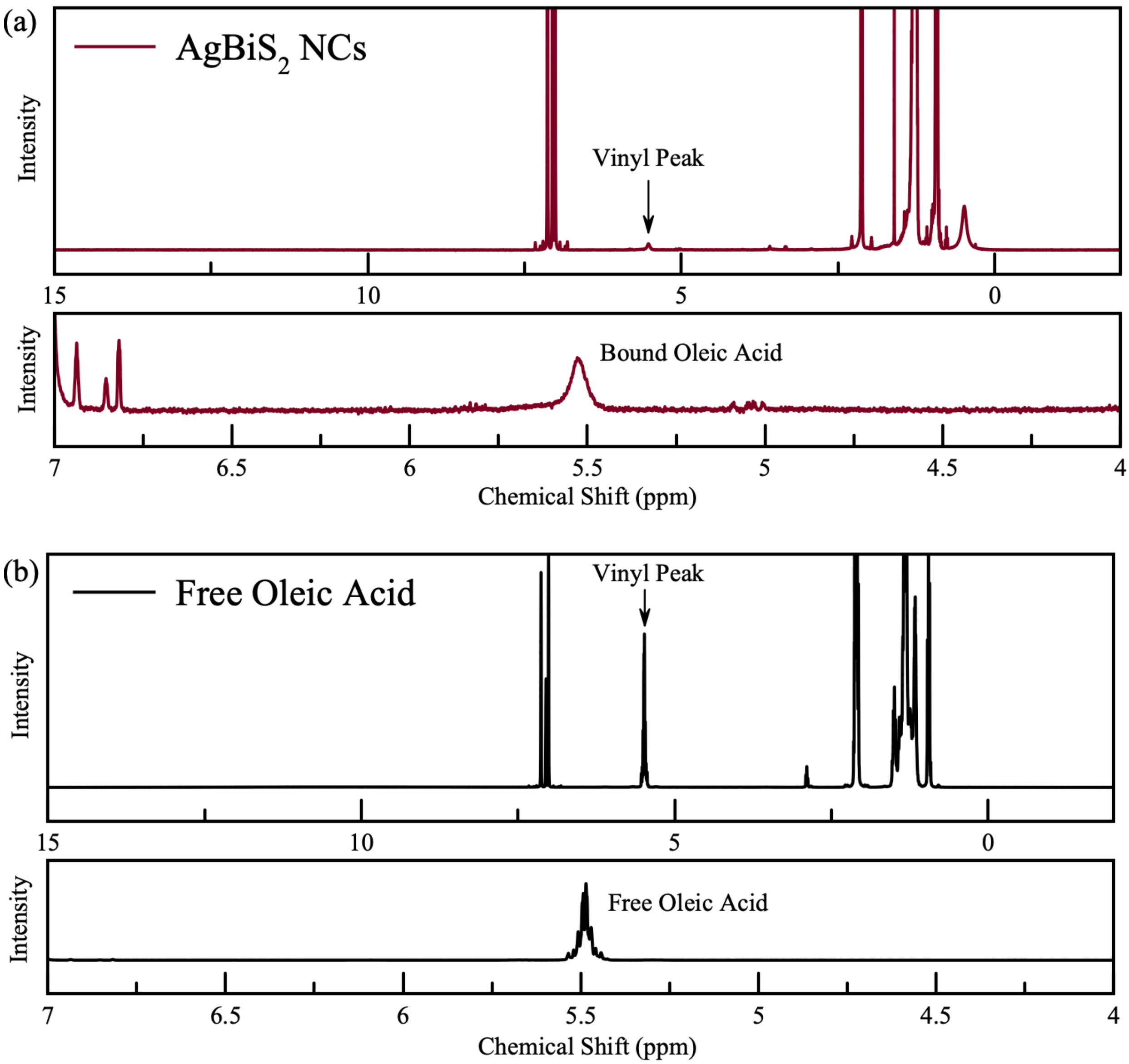

Figure S13. (a) Full (above) and zoomed in (below) ^{1}H NMR spectrum of oleic acid capped $AgBiS_2$ NCs in d-toluene (C_7D_8) showing broad resonance for the oleic acid vinylic protons and no narrow resonance, indicating all oleic acid is in bound state. (b) Full (above) and zoomed in (below) ^{1}H NMR spectrum of free oleic acid in d-toluene (C_7D_8) showing only narrow resonance.

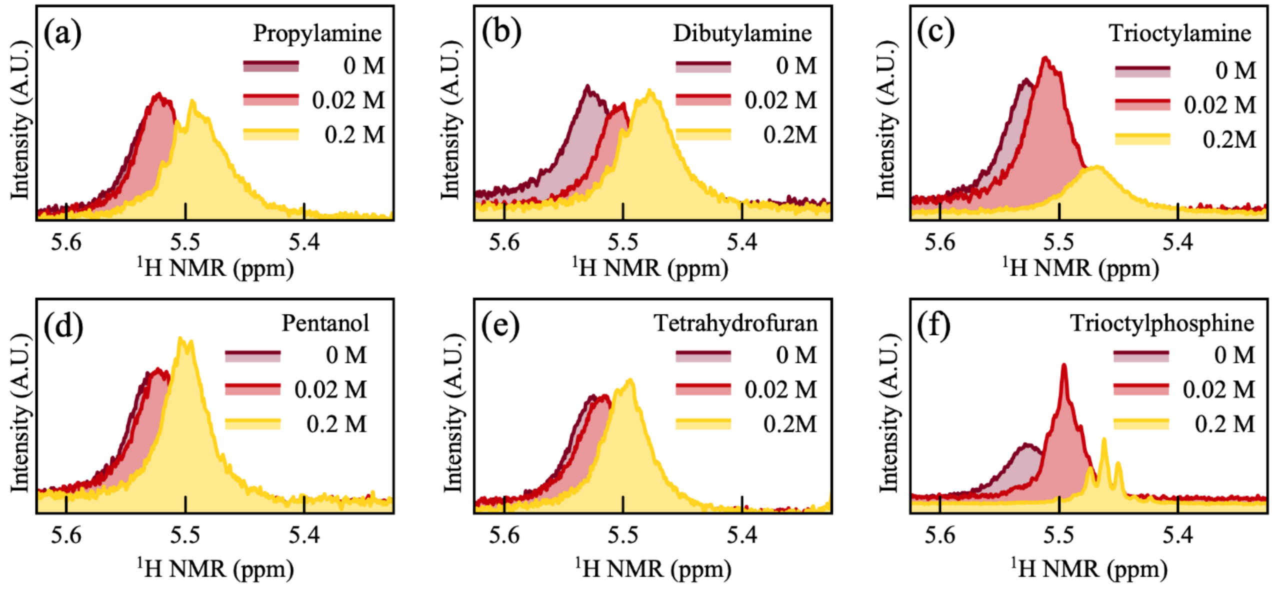

Figure S14. Vinyl Region of the ^{1}H NMR spectra of $AgBiS_2$ NCs with added Lewis bases (0.00 M (mahogany), 0.02 M (red), 0.2 M (yellow)) Appearance of sharp vinyl peak corresponds to the displacement of Ag-Oleate.

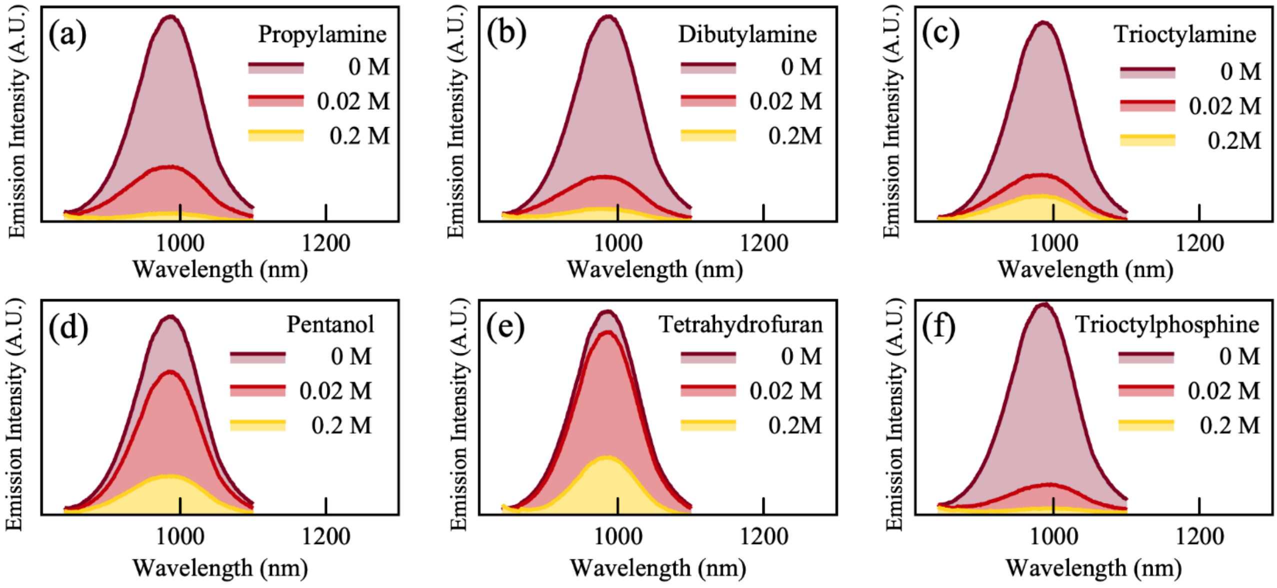

Figure S15. Normalized PL spectra of $AgBiS_2$ NCs treated with different Lewis bases. Changes to PL were monitored at 0.00 M (mahogany), 0.02 M (red) and 0.2 M (yellow). In general, increasing the concentration of Lewis bases quenches PL.

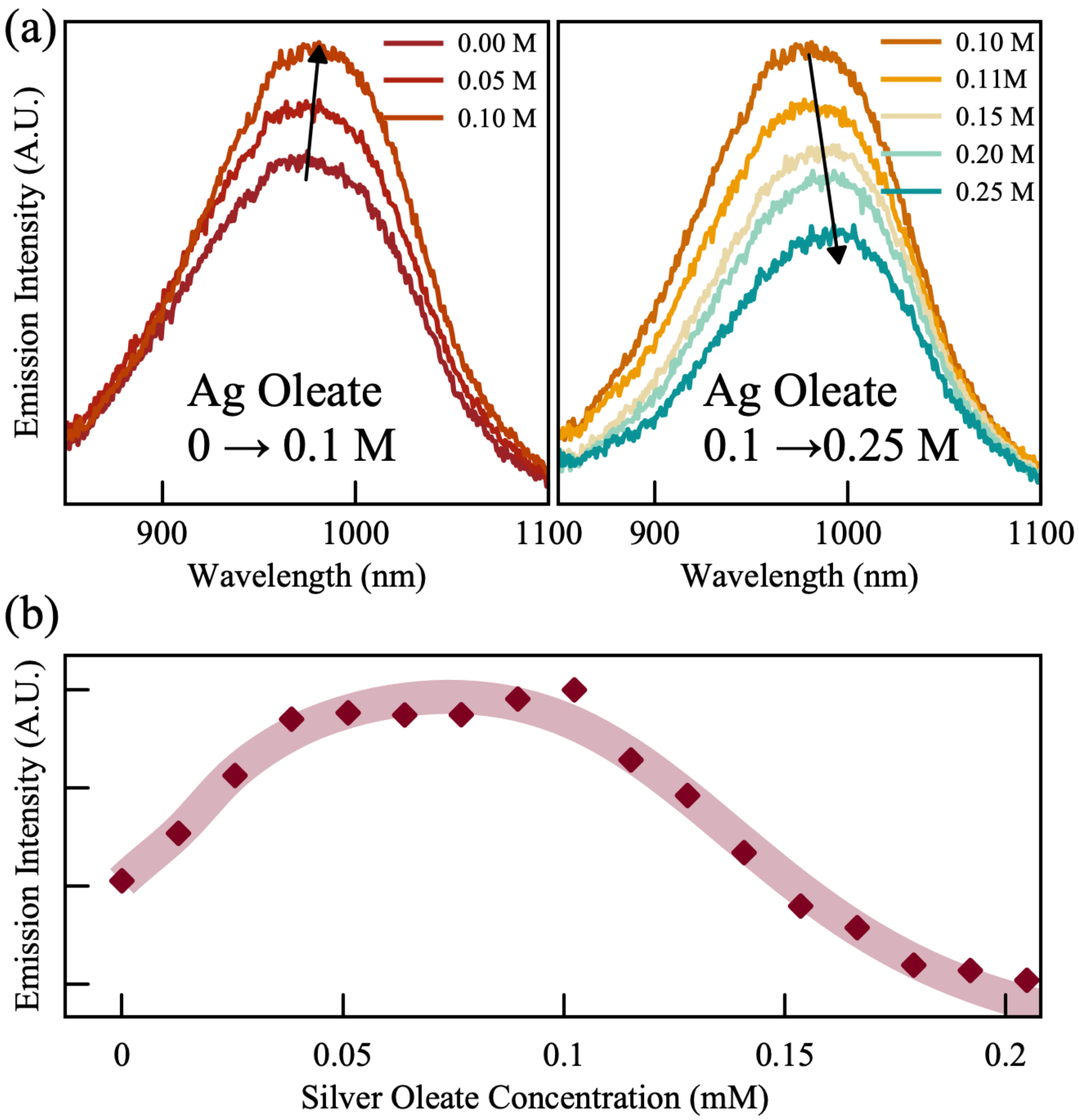

Figure S16 (a) Normalized PL spectra of 1.1:1:1 $AgBiS_2$ NCs treated with varying concentrations of Ag Oleate (left: 0.00 M to 0.10 M; right: 0.10 M to 0.25 M). The PL emission of $AgBiS_2$ NCs increases at Ag Oleate concentrations below 0.1 M due to passivation of non-radiative decay pathways (left). However, further increasing the Ag Oleate concentration reduces the PL intensity (right), attributed to cation exchange with Bi^{3+} ions in the NC lattice. (b) Normalized integrated PL intensity of 1.1:1:1 $AgBiS_2$ NCs as a function of Ag Oleate concentration (red square), with the solid line representing a fit to the data using the sum of two sigmoid functions.

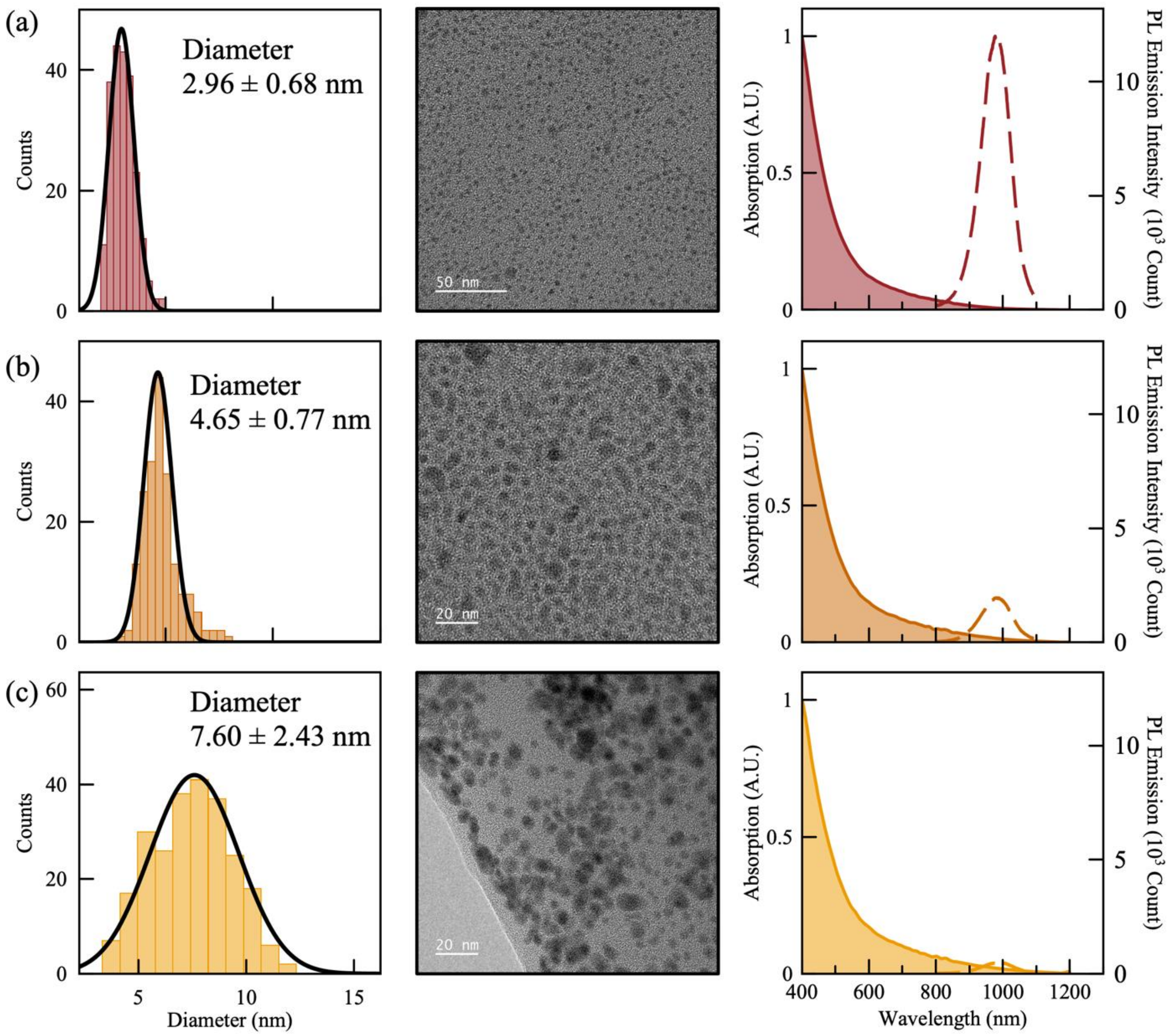

Figure S17. Size distribution histogram (Left), TEM image (Middle), absorption and PL spectra (right) of 1.2:1:1 $AgBiS_2$ NCs nanocrystals obtained from different stages of size selective precipitation. Isolated nanocrystals in the supernatant obtained after adding (a) 13 mL of acetone, (b) 10 mL of acetone, and (c) 5 mL of acetone to the crude nanocrystal product, followed by centrifugation at 11,000 rpm for 3 minutes.

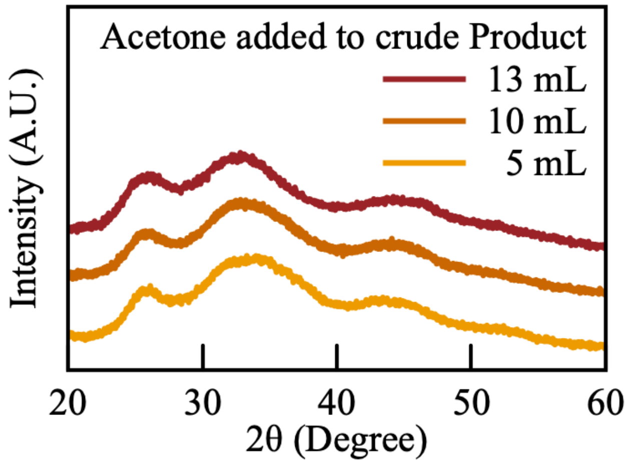

Figure S18. PXRD patterns of isolated nanocrystals in the supernatant obtained after adding 13 mL of acetone (dark red), 10 mL of acetone (orange), and 5 mL of acetone (yellow) to the crude nanocrystal product, followed by centrifugation at 11,000 rpm for 3 minutes.

Table S1 Relative amount of Ag and Bi measured using ICP-OES in different $AgBiS_2$ NC samples produced from different feeding ratio. The values are normalized in relation to Bi.

**Feeding Ratio**	**$S:Bi_{Feeding}$ = 0.7**	**$S:Bi_{Feeding}$ = 0.85**	**$S:Bi_{Feeding}$ = 1**
$Ag:Bi_{Feeding}$ = 1.3	61 : 1	3.5 : 1	1.35 : 1
$Ag:Bi_{Feeding}$ = 1.2	3.3 : 1	2.5 : 1	1.22 : 1
$Ag:Bi_{Feeding}$ = 1.1	2.4 : 1	1.1 : 1	0.9 : 1

Table S2 Stretched Exponential fitting parameters for the TRPL kinetics of $AgBiS_2$ NCs (NC) and $AgBiS_2$ /TCA complex (NC/TCA).

	**NC**	**NC/TCA**
**A_0**	1.086	4.676
**τ_k**	27.173	0.087
**β**	0.451	0.213
**$\langle\tau\rangle$**	67.104	6.204

Supplementary Note S1

This work presents the discovery of near-infrared (NIR) emission from $AgBiS_2$ NCs and the first demonstration of TTA-UC sensitization using $AgBiS_2$ NCs. This marks a significant breakthrough for the following reasons:

- **A new class of eco-friendly, easily accessible NIR sensitizers:** This work introduces a new lead-free NIR NCs, namely, $AgBiS_2$ NC, as the efficient sensitizer for TTA-UC systems. Our system can convert both NIR-I and NIR-II photons to visible photons. To the best of our knowledge, no prior study has demonstrated the use of bismuth-based NCs for photon upconversion. This expands the material platform for TTA-UC beyond the conventional toxic and carcinogenic heavy metal-based systems (e.g., Pb-, As-, or In-based materials). Although $CuInSe_2$ and InAs NCs were recently demonstrated as the NIR sensitizer for TTA-UC, they have their own limitations. $CuInSe_2$ NCs require a full optimization of stoichiometry, doping, and core/shell structure to obtain efficient TTA-UC. The use of Se also represents serious environmental and health concerns as Se is a toxic element. On the other hand, the synthesis of high-quality colloidal InAs NCs generally requires high temperature (>350 °C) and the use of *extremely toxic, highly reactive precursor* tris(trimethylsilyl)arsine (TMS_3As).[2, 3] Moreover, all indium compounds should be regarded as highly toxic[4] as they can damage the vital organs. Our $AgBiS_2$ NCs completely eliminate the toxicity risks associated with lead, selenium, arsenic and indium, making it a safe option for practical applications.[5] In addition, $AgBiS_2$ NCs can be easily synthesized (around 100°C) and demonstrate a decent UCQY without requiring additional shell coatings. Therefore, our material represents a novel, eco-friendly, and easily accessible NIR sensitizer, that is otherwise unattainable in previous demonstrations.
- **A new mechanism of NIR emission and TTA-UC.** Understanding how radiative recombination (NIR PL) and triplet sensitization occur in indirect-bandgap materials, is a very interesting, non-trivial, and important scientific question. Thus far, the radiative NIR emission from $AgBiS_2$ NCs remains unknown. And there was only *one example* showing that indirect-bandgap material (Si NCs) sensitizes molecular triplets for Visible-to-UV TTA-UC.[6] However, indirect-bandgap materials for NIR-to-visible TTA-UC remain unknown. Our work shows *the first NIR emission and triplet sensitization from $AgBiS_2$ NCs*. Therefore, our work provides a new mechanism that modulates the NIR emission and TTA-UC sensitization and offers new insights into the field of TTA-UC based on colloidal NIR QDs.

The merit of using $AgBiS_2$ NCs

Although being an indirect-bandgap material, $AgBiS_2$ NCs exhibit an unusually high absorption coefficient. Remarkably, $AgBiS_2$ NCs show absorption coefficients that are 5–10 times higher than those of many common solar cell materials,[7] including PbS, InP, GaAs, and organic lead halide perovskites (Figure S19). For example, the absorption coefficient of $AgBiS_2$ at 800 nm is measured to be ~ 1.2×10^5 cm^{-1}, which exceeds that of many high-performance direct bandgap materials including GaAs (1×10^4 cm^{-1}), CdTe (5×10^4 cm^{-1})[8] and PbS (1.1×10^4 cm^{-1}).[9] Therefore, our $AgBiS_2$ NC is indeed a unique material for NIR-to-Visible photon upconversion.

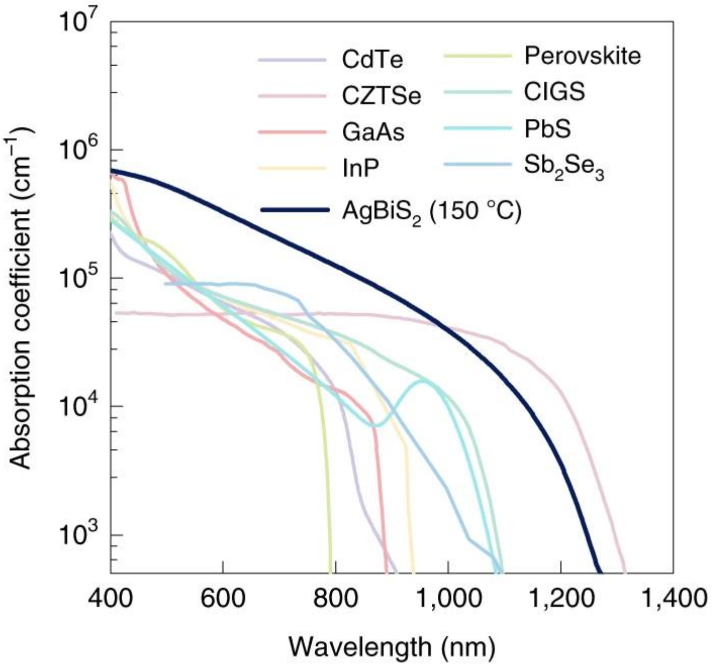

Figure S19. Absorption coefficient of different common photovoltaic materials from 400 nm to 1400 nm at 300K. Black solid line represents the absorption coefficient of $AgBiS_2$ NCs.[7]

Supplementary Note S2

To estimate the rate and efficiency of the TET-1 process (NC to TCA), we applied a previous model[13] to fit the TRPL kinetics (after 1 ns) with stretched exponential functions,

$$A(t) = A_0 e^{-(\frac{t}{\tau_K})^{\beta}} \quad \text{(eq. S1)}$$

The average lifetime of each TRPL kinetics trace can then be calculated based on,

$$\langle\tau\rangle = \frac{\tau_K}{\beta}\Gamma(\frac{1}{\beta}) \quad \text{(eq. S2)}$$

Where Γ is the gamma function. With the measured average PL lifetime of $AgBiS_2$ NCs ($\langle\tau_{AgBiS_2}\rangle$) and $AgBiS_2$-TCA NCs ($\langle\tau_{AgBiS_2\text{-}TCA}\rangle$), the apparent TET-1 rate (k_{TET}) and efficiency (η) are calculated as,

$$k_{TET} = \frac{1}{\langle\tau_{AgBiS_2-TCA}\rangle} - \frac{1}{\langle\tau_{AgBiS_2}\rangle} \quad \text{(eq. S3)}$$

$$\eta = \frac{\kappa_{TET}}{(\frac{1}{\tau_{AgBiS_2}})} \quad \text{(eq. S4)}$$

The apparent TET-1 rate is determined as 0.146 ns^{-1}, with a TET-1 efficiency of 90.8% in $AgBiS_2$-TCA complex. Fitting parameters are shown in Table S2.

Supplementary Note S3

The UCQY under 980 nm excitation (0.8%) is significantly lower than that observed under 808 nm excitation but is substantially higher than previously reported upconversion efficiencies for 980 to 560 nm processes.[10] This significantly lower UCQY at 980 nm can be attributed to two key factors. First, the absorption coefficient of $AgBiS_2$ NCs at 980 nm is approximately four times lower than at 808 nm, resulting in a higher threshold intensity for saturating the two-photon process in TTA-UC. Second, efficient triplet energy transfer (TET) requires a favorable energy alignment between the NC exciton (~1.28 eV), the TCA triplet (~1.3 eV), and the rubrene triplet (~1.14 eV). Under 808 nm excitation (1.54 eV), the minimal energy difference between the NC exciton and TCA triplet already limits TET-1 efficiency. At 980 nm (1.26 eV), the driving force becomes even weaker, further suppressing TET-1.

Interestingly, despite this unfavorable energy alignment, $AgBiS_2$ NCs exhibit relatively high UCQY under 980 nm excitation compared to other NIR-II NC systems.[11, 12] We hypothesize that phonon coupling within $AgBiS_2$ NCs may assist excitons in overcoming the energy barrier for TET-1 with TCA. This phonon-assisted mechanism likely accounts for the observed upconversion performance. Additionally, as no UC emission is observed under 980 nm excitation in the absence of TCA functionalization, direct energy transfer from NCs to rubrene (bypassing TCA) can be excluded. These results warrant the need for further exploration and optimization of $AgBiS_2$ sensitized 980 nm TTA-UC.

Supplementary Note S4

The enhanced UCQY observed at 200 K can be understood through the interplay of multiple factors. The upconversion quantum yield (UCQY) is given by the expression:

$$\Phi'_{UC} = \Phi_{TET-1}\Phi_{TET-2}\Phi_{TTA}\Phi_{PL} \text{ (eq. S5)}$$

Here, Φ_{TET-1} and Φ_{TET-2} represent the triplet energy transfer (TET) quantum efficiencies from QDs to the transmitter and from the transmitter to the annihilator, respectively. Φ_{TTA} is the singlet generation efficiency via triplet–triplet annihilation (TTA), and Φ_{PL} is the photoluminescence quantum efficiency of the annihilator. Each of these terms can be impacted differently by temperature. At lower temperatures, Φ_{TET-1} may improve due to suppressed non-radiative recombination in the nanocrystals (NCs), while Φ_{TET-2} is enhanced by the extension of the triplet lifetime of the transmitters. However, Φ_{TTA} may decrease as a result of suppressed triplet diffusion, and Φ_{PL} is likely to increase due to reduced thermal quenching.

This interplay of factors results in the overall enhancement of UCQY at 200 K. However, the exact contributions of each step and their temperature dependence remain unclear and warrant further investigation. This phenomenon is discussed here to provide additional context, and future studies will aim to conduct detailed temperature-dependent measurements to unravel the underlying mechanisms.

Supplementary Note S5

The TET-2 process from TCA to rubrene is a bimolecular process, depending on both the rubrene concentration ([Rub]) and the triplet lifetime of the TCA ligand (τ_{TCA}). We can then estimate the efficiency of TET-2 (Φ_{TET-2}) from the following equation[14] :

$$\Phi_{TET-2} = 1 - \frac{1}{1+k_{TET-2}\tau_{TCA}[Rub]} \text{ (eq. S6)}$$

We assume the bimolecular transfer rate from TCA to rubrene (k_{TET-2}) to be $1 \times 10^8 M^{-1}s^{-1}$ from similar systems.[15, 16] With the rubrene concentration of 20 mM and an average ^{3}TCA* lifetime of 4.7 µs, the Φ_{TET-2} in our system is estimated to be 90.3 %. From eq.1, we can approximate a Φ_{TTA} of 6.6 %, which is slightly higher than the efficiency given purely by the spin statistics – 5.55 % (singlet, triplet, and quintet in a 1:3:5 ratio, max 50%).

Φ_{UC} (max 50%)	Φ_{TET-1}	Φ_{TET-2}	Φ_{TTA} (max 50%)	Φ_{PL}
5.3 %	90.8 %	90.3 %	6.6 %	98.0 %

We rule out the possibility of direct triplet energy transfer (TET) between the $AgBiS_2$ NCs and rubrene based on two observations:

1. No photoluminescence (PL) quenching was observed when NCs and Rubrene were mixed in toluene.
2. No detectable upconversion emission was observed when the NC surface was not functionalized with TCA.

Supplementary Note S6

Emission measurements conducted within a shared optical setup using substantially differing excitation wavelengths—such as those in the visible and near-infrared (NIR) regions—are subject to chromatic aberration, which can result in variations in focal length and consequently the beam diameter at the sample.

During our measurement of both UCQY, we have confirmed that the beam size of both 808 nm and 405 nm lasers have a comparable diameter on the sample using the knife edge method (Figure S20). Therefore, the effect of chromatic aberration in our optical setup is negligible.

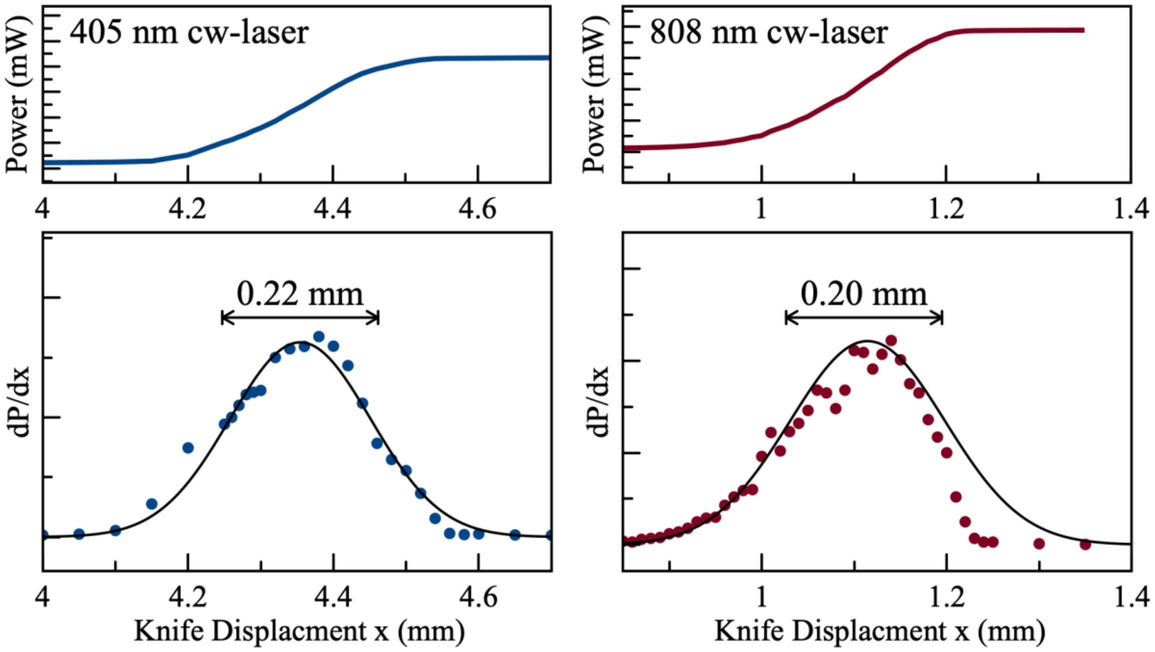

Figure S20. Beam size determination for 405 nm (blue) and 808 nm (red) cw-lasers using the knife-edge method. The upper panels show the measured power as a function of knife-edge displacement, while the lower panels display the first derivative of the power. Solid black lines represent Gaussian fits to the data. Beam sizes for the cw-lasers are determined from the full-width at half-maximum (FWHM) of the Gaussian fits. The effect of chromatic aberration in our optical setup appears to be negligible.

Supplementary References

(1) Liang, W. F.; Nie, C. M.; Du, J.; Han, Y. Y.; Zhao, G. H.; Yang, F.; Liang, G. J.; Wu, K. F. Near-infrared photon upconversion and solar synthesis using lead-free nanocrystals. *Nat. Photonics* **2023**, *17* (4), 346

(2) Sun, R.; Zang, J.; Lai, R.; Yang, W.; Ji, B. Near-Infrared-to-Visible Photon Upconversion with Efficiency Exceeding 21% Sensitized by InAs Quantum Dots. *J. Am. Chem. Soc.* **2024**, *146* (26), 17618-17623

(3) Sun, R. J.; Zang, J. Y.; Lai, R. C.; Yang, W. X.; Ji, B. T. Near-Infrared-to-Visible Photon Upconversion with Efficiency Exceeding 21% Sensitized by InAs Quantum Dots. *J. Am. Chem. Soc.* **2024**, *146* (26), 17618-17623

(4) Barnabas, M. J.; Parambadath, S.; Mathew, A.; Park, S. S.; Vinu, A.; Ha, C. S. Highly efficient and selective adsorption of In3+ on pristine Zn/Al layered double hydroxide (Zn/Al-LDH) from aqueous solutions. *J. Solid State Chem.* **2016**, *233*, 133-142

(5) Chen, B. J.; Zhang, C. Y.; Wang, W. N.; Chu, Z. Y.; Zha, Z. B.; He, X. Y.; Zhou, W.; Liu, T.; Wang, H.; Qian, H. S. Ultrastable AgBiS2 Hollow Nanospheres with Cancer Cell-Specific Cytotoxicity for Multimodal Tumor Therapy. *Acs. Nano* **2020**, *14* (11), 14919-14928
(6) Xia, P.; Raulerson, E. K.; Coleman, D.; Gerke, C. S.; Mangolini, L.; Tang, M. L.; Roberts, S. T. Achieving spin-triplet exciton transfer between silicon and molecular acceptors for photon upconversion. *Nat. Chem.* **2020**, *12* (2), 137-144
(7) Wang, Y. J.; Kavanagh, S. R.; Burgués-Ceballos, I.; Walsh, A.; Scanlon, D.; Konstantatos, G. Cation disorder engineering yields $AgBiS_2$ nanocrystals with enhanced optical absorption for efficient ultrathin solar cells. *Nat Photonics* **2022**, *16* (3), 235
(8) Treharne, R. E.; Seymour-Pierce, A.; Durose, K.; Hutchings, K.; Roncallo, S.; Lane, D. Optical Design and Fabrication of Fully Sputtered CdTe/CdS Solar Cells. *J Phys Conf Ser* **2011**, *286*,
(9) Palik, E. D. *Handbook of optical constants of solids*; Academic Press, 1985
(10) Jiang, L. H.; Miao, X.; Zhang, M. Y.; Li, J. Y.; Zeng, L.; Hu, W.; Huang, L.; Pang, D. W. Near Infrared-II Excited Triplet Fusion Upconversion with Anti-Stokes Shift Approaching the Theoretical Limit. *J. Am. Chem. Soc.* **2024**, *146* (15), 10785-10797
(11) Huang, Z.; Li, X.; Mahboub, M.; Hanson, K. M.; Nichols, V. M.; Le, H.; Tang, M. L.; Bardeen, C. J. Hybrid Molecule-Nanocrystal Photon Upconversion Across the Visible and Near-Infrared. *Nano Lett.* **2015**, *15* (8), 5552-5557
(12) Wu, M.; Lin, T. A.; Tiepelt, J. O.; Bulovic, V.; Baldo, M. A. Nanocrystal-Sensitized Infrared-to-Visible Upconversion in a Microcavity under Subsolar Flux. *Nano Lett.* **2021**, *21* (2), 1011-1016
(13) Jin, T.; Lian, T. Trap state mediated triplet energy transfer from CdSe quantum dots to molecular acceptors. *J. Chem. Phys.* **2020**, *153* (7), 074703
(14) Gray, V.; Drake, W.; Allardice, J. R.; Zhang, Z.; Xiao, J.; Congrave, D. G.; Royakkers, J.; Zeng, W.; Dowland, S.; Greenham, N. C.; et al. Triplet transfer from PbS quantum dots to tetracene ligands: is faster always better? *J. Mater. Chem. C Mater.* **2022**, *10* (43), 16321-16329
(15) Cheng, Y. Y.; Fückel, B.; Khoury, T.; Clady, R. G. C. R.; Tayebjee, M. J. Y.; Ekins-Daukes, N. J.; Crossley, M. J.; Schmidt, T. W. Kinetic Analysis of Photochemical Upconversion by Triplet−Triplet Annihilation: Beyond Any Spin Statistical Limit. J. Phys. Chem. Lett. **2010**, *1* (12), 1795-1799
(16) Schmidt, T. W.; Castellano, F. N. Photochemical Upconversion: The Primacy of Kinetics. *J. Phys. Chem. Lett.* **2014**, *5* (22), 4062-4072
